# SLIMM: Slice localization integrated MRI monitoring

**DOI:** 10.1016/j.neuroimage.2020.117280

**Published:** 2020-08-24

**Authors:** Yao Sui, Onur Afacan, Ali Gholipour, Simon K. Warfield

**Affiliations:** a Computational Radiology Laboratory, Boston Children’s Hospital, Boston, MA, USA; b Harvard Medical School, Boston, MA, USA

**Keywords:** Prospective motion monitoring, fMRI, Slice-to-volume image registration, Self-navigated motion measurement

## Abstract

Functional MRI (fMRI) is extremely challenging to perform in subjects who move because subject motion disrupts blood oxygenation level dependent (BOLD) signal measurement. It has become common to use retrospective framewise motion detection and censoring in fMRI studies to eliminate artifacts arising from motion. Data censoring results in significant loss of data and statistical power unless the data acquisition is extended to acquire more data not corrupted by motion. Acquiring more data than is necessary leads to longer than necessary scan duration, which is more expensive and may lead to additional subject non-compliance. Therefore, it is well established that real-time prospective motion monitoring is crucial to ensure data quality and reduce imaging costs. In addition, real-time monitoring of motion allows for feedback to the operator and the subject during the acquisition, to enable intervention to reduce the subject motion. The most widely used form of motion monitoring for fMRI is based on volume-to-volume registration (VVR), which quantifies motion as the misalignment between subsequent volumes. However, motion is not constrained to occur only at the boundaries of volume acquisition, but instead may occur at any time. Consequently, each slice of an fMRI acquisition may be displaced by motion, and assessment of whole volume to volume motion may be insensitive to both intra-volume and inter-volume motion that is revealed by displacement of the slices. We developed the first slice-by-slice self-navigated motion monitoring system for fMRI by developing a real-time slice-to-volume registration (SVR) algorithm. Our real-time SVR algorithm, which is the core of the system, uses a local image patch-based matching criterion along with a Levenberg-Marquardt optimizer, all accelerated via symmetric multi-processing, with interleaved and simultaneous multi-slice acquisition schemes. Extensive experimental results on real motion data demonstrated that our fast motion monitoring system, named Slice Localization Integrated MRI Monitoring (SLIMM), provides more accurate motion measurements than a VVR based approach. Therefore, SLIMM offers improved online motion monitoring which is particularly important in fMRI for challenging patient populations. Real-time motion monitoring is crucial for online data quality control and assurance, for enabling feedback to the subject and the operator to act to mitigate motion, and in adaptive acquisition strategies that aim to ensure enough data of sufficient quality is acquired without acquiring excess data.

## 1. Introduction

Head motion adversely affects data quality in functional magnetic resonance imaging (fMRI), leading to distorted images, biased analyses, and increased cost due to the need for repeating scans (Kim et al., 1999; Lanka and Deshpande, 2019; Muraskin et al., 2013; Murphy et al., 2007; Power et al., 2014; 2015; Schulz et al., 2014).

Systematic bias in fMRI analyses due to residual motion artifacts, such as spurious reduction of long-range functional connectivity and increased strength of short-range functional connectivity, has been well documented (Deen and Pelphrey, 2012; Power et al., 2012). Motion effects in fMRI are subtle and cannot be easily evaluated during acquisition to ensure data of sufficient quality are obtained for robust fMRI analysis.

### 1.1. Background and significance

fMRI is routinely acquired as time series of stacks of 2D gradient-echo, echo planar imaging (EPI) slices (Stehling et al., 1991) that generate a blood oxygenation level dependent (BOLD) contrast by measuring the $T_{2}^{*}$ relaxation of nuclei, which changes by the concentration of deoxyhemoglobin. The BOLD signal is thus used to measure neural activity due to the hemodynamic response. Task-based and resting state fMRI are both widely used, and range in duration from around 5 min to over an hour. In fact, during the relatively long fMRI acquisition time, head motion is common, even for healthy and cooperative adult subjects, as is physiological motion associated with respiration and cardiac pulsation. Research has shown that sub-millimeter head movements systematically degrade the accuracy of fMRI analyses (Power et al., 2012; Satterthwaite et al., 2012; Schulz et al., 2014; Van Dijk et al., 2012).

Much effort has been made in alleviating the effects of head motion artifacts on fMRI analysis by means of post-acquisition processing (Ciric et al., 2017; Power et al., 2012; 2015; Satterthwaite et al., 2013). However, since these techniques are retrospective, they require that sufficient motion-free data is collected. To ensure that the acquired data are sufficient for analysis, a typical strategy, in the face of unknown motion-induced data loss, is to acquire additional data (i.e., over-scanning). Data that are corrupted by motion are then excluded by retrospective data censoring (Ciric et al., 2017). Although such a strategy increases the likelihood of collecting sufficient data, it is highly inefficient, and significantly increases imaging time and costs. Ultimately, it does not guarantee that data of sufficient quality is acquired for every subject in a cohort. This strategy renders imaging studies of challenging populations particularly inefficient and costly. As MRI resources are often expensive and limited, it is desirable to optimize the length of data acquisition, and to acquire data without motion as much as possible. Unnecessarily long scans also increase the burden of imaging on patients and vulnerable cohorts. The increased imaging time due to patient motion has been estimated to cost clinical and research studies over $115,000 per scanner per year, and $1.4B per year in the United States alone (Andre et al., 2015). In resting state fMRI alone, motion increases the scan times and the associated costs by over 57% (Dosenbach et al., 2017).

The issues listed above affect the design of all fMRI studies, but are significantly more prominent in fMRI studies of non-cooperative subjects, such as infants, toddlers, and patients who have difficulty in avoiding moving their heads during fMRI scans. In a pediatric patient cohort studied by Dosenbach et al. (2017), over 50% of the fMRI data were reported to be unusable under their data censoring criteria (frame displacement > 0.2 mm). The high rate of data loss in pediatric fMRI suggests it would be beneficial to monitor for motion during the acquisition itself. This is known as real-time motion monitoring. Real-time motion monitoring allows the scanner operators to intervene, to provide feedback to subjects when they are moving, and to ensure that data acquisition may be extended until a sufficient amount of motion-free data is acquired. Real-time motion monitoring enables the design of adaptive scanning strategies based on the analysis of motion and their effect on fMRI quality. Research has shown that immediate feedback to the subject, such as direction from the operator, or motion-induced modification of stimuli presentation, substantially improves the time for which children hold still during fMRI (Dosenbach et al., 2017; Greene et al., 2018).

### 1.2. State-of-the-art in MRI motion monitoring

A variety of techniques can be used for motion monitoring in fMRI (Zaitsev et al., 2017), including optical motion tracking systems (Forman et al., 2010; Schulz et al., 2014; Zaitsev et al., 2006), systems based on active markers (Muraskin et al., 2013), navigator-based methods (Ehman and Felmlee, 1989; White et al., 2010), and image alignment-based methods (Friston et al., 1995; Jenkinson et al., 2002; Thesen et al., 2000). These techniques, however, have limitations. Navigator-based methods (Ehman and Felmlee, 1989; White et al., 2010) that insert k-space or image navigators into the imaging sequences at different time points for motion measurement, are highly dependent on the imaging platform and typically require constraints on protocol parameters, such as repetition and echo times (TR and TE), which may adversely affect the acquisition time and signal-to-noise ratio (SNR).

Optical trackers (Forman et al., 2010; Schulz et al., 2014; Zaitsev et al., 2006) often require markers attached to the patient’s head and a direct line of sight between camera and markers (or face) must be maintained. These systems can be difficult to set up or incompatible with the visual presentation of fMRI stimulus on a set of goggles, or fixation on a visual cross on a mirror during resting state fMRI acquisitions. Moreover, methods with external hardware and navigators are generally platform-dependent, increase costs, and may raise safety concerns. Attaching markers on skin may irritate or disturb infants and young children.

Self-navigated methods do not require additional navigator data or hardware as they rely on the acquired data only to estimate motion (Dosenbach et al., 2017; Greene et al., 2018; Thesen et al., 2000). Among these, a method known as Prospective Acquisition Correction (PACE) (Thesen et al., 2000), uses volume-to-volume registration (VVR) to infer motion parameters between fMRI volumes. PACE correction estimates the motion by calculating the registration of one volume to another volume, and is then able to steer to the position of the previous volume at the time of the next volume acquisition. PACE assumes there is no motion between the volume that was acquired and the next volume to be acquired. Since it can only correct for motion once per volume, it is unable to correct for the displacement of slices that occurs when motion is entirely within a volume, or when different motion patterns occur for slices that are part of more than one volume. Therefore, PACE is not effective for the fast and frequent motion that commonly happens with non-cooperative subjects (Lanka and Deshpande, 2019). A more recent technique in this category is FIRMM (framewise integrated real-time MRI monitoring) (Dosenbach et al., 2017; Greene et al., 2018), which is a self-navigated technique for motion monitoring during fMRI with real-time performance (i.e., motion is measured within the time period of the next volume acquisition). Motion monitoring in FIRMM also relies on a VVR strategy.

Nevertheless, fMRI is acquired as a sequence of 2D slices, and motion happens at the slice level. Techniques that monitor fMRI motion at the volume level have limited temporal resolution and are insufficiently sensitive to intra-volume motion. The limitations of VVR-based strategies have been investigated in a series of studies. Kim et al. (1999) were the first to develop and show the added value of retrospective slice-level motion correction via slice-to-volume registration (SVR) over VVR. Beall and Lowe (2014b) developed a retrospective slicewise motion correction method for fMRI data, known as the SLOMOCO, and quantitatively assessed the superiority of the correction at the slice level over that at the volume level in (Beall and Lowe, 2014a). Compared to VVR-based techniques, motion correction using retrospective SVR followed by image reconstruction has shown significantly improved results in a range of MRI applications including diffusion-weighted imaging (DWI) of non-cooperative patients, e.g. (Bastiani et al., 2019; Hutter et al., 2018; Marami et al., 2016; 2019), fetal brain MRI (Alansary et al., 2017; Ebner et al., 2019b; Gholipour et al., 2010; Kainz et al., 2015; Marami et al., 2017), fetal cardiac MRI (van Amerom et al., 2019; Lloyd et al., 2019), and body MRI (Ebner et al., 2019a; Kurugol et al., 2017).

All of the above-mentioned techniques, however, are based on *retrospective* SVR. Despite the proven advantages of SVR over VVR to estimate and correct for slice-level motion, there is currently no real-time motion monitoring system based on SVR. This is mainly due to the technical challenges in achieving fast and yet accurate and reliable SVR in ultra-fast sequences such as fMRI. In this paper, we report the first development and evaluation of a *real-time* slice-by-slice motion monitoring algorithm for fMRI. Real-time in this context means that motion is measured within the time period of the next slice acquisition during fMRI, which is in the order of 40–80ms.

To achieve real-time performance, our technique extracts a set of local image features and incorporates a feature selection scheme to create a similarity metric for SVR. Accelerated optimization is achieved through finite difference computation of the similarity gradients with respect to transformation parameters within a Levenberg-Marquardt (LM) optimization framework (Levenberg, 1944; Marquardt, 1963).

The experimental results reported in this paper are based on fMRI data sets with observed patterns of motion. Our analysis and experimental results show that our feature selection and gradient computation schemes led to improved registration accuracy and an accelerated optimization process that has enabled real-time SVR for a fast, self-navigated motion monitoring system.

### 1.3. Contributions

This work has two main contributions:

1. A *real-time* slice-to-volume image registration algorithm in which a local image feature extraction and selection scheme is leveraged to compute a (dis)similarity metric for improved registration performance; and a parallelized gradient computation method accelerates the optimization process for image registration. The technique leverages interleaved and simultaneous multi-slice acquisition schemes to improve registration stability and robustness.
2. A real-time slice-by-slice motion monitoring system, called SLIMM for Slice-Localization Integrated MRI Monitoring, is established with the proposed slice-to-volume registration algorithm. To the best of our knowledge, this is the first work to perform self-navigated slice-by-slice motion monitoring in real-time for fMRI. Extensive experimental results on fMRI data sets with observed patterns of motion show that SLIMM is able to process 27.7 slices per second with motion estimation errors under 0.8 degrees in rotation and, on average, under 0.75 mm (1/4 voxel size) in translation. The achieved performance is satisfactory for slice-by-slice motion monitoring in fMRI.

The remainder of this paper is organized as follows: The material and methods are presented in Section 2. The results are shown in Section 3. We discuss our results and concluding observations in Section 4.

## 2. Methods

The purpose of the approach developed and presented here is to prospectively measure the subject motion during fMRI slice acquisitions via a real-time SVR algorithm, enabling the data acquisition duration to be dynamically adjusted to be shorter or longer, and enabling the motion information to be presented to the scan operator and the subject during the data acquisition. In this work we used 2D EPI sequences for fMRI experiments, with simultaneous multi-slice acquisition, where each slice is acquired in around 80ms. In order for the real-time motion monitoring to use every slice, the alignment of the slice to the reference volume should be completed in less time than required to construct the next slice (<= 80 ms). As each fMRI slice was acquired, we registered each slice to a reference volume using our real-time SVR algorithm. Motion is thus measured by the change in the position of the acquired slice before and after SVR. Online motion measurements are then fed back to scanner operators to make immediate decisions on continuing or stopping scans, or to enable them to direct the subject to hold still, or to modify the stimuli seen by the subject to indicate excessive motion is occurring, again enabling the subject to reduce their motion.

### 2.1. Our algorithm and theory

In SVR, a reference 3D volumetric (moving) image is transformed in 3D space to align to an input (fixed) 2D slice image. Therefore, given a 2D slice image *I* and a 3D volumetric image *J*, SVR can be formulated by a minimization problem:

(1) $$\theta^{*}=\text{arg }\underset{\theta }{\text{min}}f\left(I,T_{n}(J;\theta )\right)+\lambda r(\theta ),$$

where the function *f*(·, ·) serves as a matching criterion that measures the similarity between the reference and source images; *T*_*n*_(·; ***θ***) transforms the input volumetric image to the space of the slice image that has the motion state ***θ*** and then outputs the *n*-th oblique slice; *r*(***θ***) defines a regularization term (i.e., prior knowledge on ***θ***) that constrains the transformation; and *λ >* 0 is a weight parameter balancing the cost between the similarity and the regularization terms. The solution θ* represents the spatial transformation between the slice and the volume in a common space.

#### 2.1.1. Motion representation

When a subject is deliberately and carefully holding still, there are still several physiological sources of motion or apparent motion. These include small magnitude displacements such as pulsation due to the cardiac cycle, and motion induced at the head due to breathing, together with dynamic modulations of the static field induced by respiration and motion of other body parts that induce phase encoding artifacts that disrupt spatial coding. There may also be referred motion at the head associated with moving the legs or arms. The head often moves if a subject swallows. Thus, even in the absence of overt movement, there are a number of sources of motion or apparent motion that may be characterized by nonrigid motion. Typically no motion monitoring is carried out for these types of motion, and retrospective motion correction is used to correct for cardiac and respiratory related effects.

In addition to these physiological sources, subjects may displace their head in a rigid body fashion, undergoing certain types of rotation and translation. It is this rigid body motion imposed on top of smaller magnitude physiological motion sources, that is sought to be measured by volume to volume monitoring with techniques such as PACE and FIRMM, and retrospective correction. Similarly, our goal is to monitor the rigid body motion that may displace the position of each source.

Head motion is therefore modeled as a rigid body transformation in our SVR algorithm with 6 degrees of freedom in 3D space (Birkfellner et al., 2007; Fogtmann et al., 2014; Gholipour et al., 2011; Jiang et al., 2007; Kainz et al.,2015; Marami et al., 2016), with the parameters ***θ*** = [*α, β, γ, t*_*x*_*, t*_*y*_*, t*_*z*_]^*T*^ that represent the motion state of the subject head in the scanner (world) coordinate system; where *α, β*, and *γ* denote the rotation angles about the axes-*x, y*, and *z*, and *t*_*x*_*, t*_*y*_, and *t*_*z*_ denote the translations along the axes-*x, y*, and *z*, respectively. In neuroimaging, padding and soft restraints are put around the patient’s head in head coils to restrict motion and protect the head in particular for children and infants. Since this limits the range of subject motion, the rigid-body assumption is sufficiently expressive to represent patient motion, considering that the interaction effects of field in-homogeneities and geometric distortions present but yield second order (Beall and Lowe, 2014a). In motion measurement, we compute the motion state ***θ***_*t*_ of slice *I*_*t*_ acquired at time *t* by registering slice *I*_*t*_ to the reference volume (with a prior motion state ***θ***_0_). The absolute motion of the slice *I*_*t*_ against the reference volume is thus computed as Δ***θ***_*t*_ = ***θ***_*t*_ − ***θ***_0_. Correspondingly, head movement at the *t*th slice is measured by Δ***θ***_*t*_ – Δ***θ***_*t*−1_.

#### 2.1.2. Matching criterion

The matching criterion, also known as the similarity metric, quantifies how well (accurately) a slice is registered to the reference volume. The choices of the matching criterion depend on the nature of the problem. For the matching criterion in image registration, two approaches are mainly adopted: iconic and geometric (Akselrod-Ballin et al., 2011; Ferrante and Paragios, 2017). Iconic criteria employ voxel intensities to quantify the similarity (Bhagalia et al., 2009; Elen et al., 2010; Ferrante and Paragios, 2013; Fogtmann et al., 2014; Jiang et al., 2007; Kainz et al., 2015; Marami et al., 2016), whereas geometric criteria exploit the correspondences between anatomical locations or salient image regions (Bay et al., 2006; Besl and McKay, 1992; Dalal and Triggs, 2005; Lowe, 1999; Nir et al., 2014).

As shown in Eq. (1), the similarity between the slice and the reference volume is computed from the similarity between the slice and a second slice formed from a plane of the volume according to ***θ*** (position and orientation). With the input slice *I* and the extracted slice *I*′, the similarity *f*(·, ·) can be evaluated. In this work, we build a matching criterion based on the Euclidean distance between local image patches. There are two advantages to be gained from this matching criterion when minimizing *f*(·, ·) in the registration: first, it poses a nonlinear, unconstrained least squares problem that can be solved by gradient descent-based algorithms scaled by parallel computing techniques; and second, it can be easily extended to other metrics, e.g., the global normalized correlation metric by zeroing the means and rescaling the two slices to unit $l_{2}\text{-norm}$, respectively.

The gradient descent-based algorithms that find transformation parameters by maximizing the matching criterion, benefit from data being independent and identically distributed (i.i.d), because 1) if the data samples are strongly correlated to each other, the gradient used to update the objective would be dominated by only a few representative samples (e.g., centers of the clusters); and 2) if the data samples are from different distributions, they would compete against each other when computing the gradient and may weaken the gradient magnitude. These can lead to slow convergence or convergence to local optima. It is, however, unreasonable to assume that pixel intensities of the slices are i.i.d, because of their strong correlations to their neighboring pixels. In contrast, densely sampled local image patches can be considered to be i.i.d based on the inherent structures that have been demonstrated to exist in MR images (Plenge et al., 2013), and also in natural images (Sui and Zhang, 2016). This is known as the local self-similarity in MR images, and as the local structure in natural images. Motivated by this, we define our matching criterion based on distances of local image patches sampled from the moving and target image slices:

(2) $$f\left(I,I^{\prime }\right)=\sum_{k\in S}{\left\|p_{k}(I)-p_{k}\left(I^{\prime }\right)\right\|}_{2}^{2},$$

where the patch extractor operation *p*(·) densely samples, with overlap, the local image patches from input slice. Those patches are indexed by the set S. Since the densely sampled local patches reside on a low-dimensional subspace or manifold due to their strong local correlations, they can be approximated by samples drawn from an independent and identical multivariate Gaussian distribution.

Certain slice images contain a large number of homogeneous patches, e.g., patches from the background. Using such patches that have many correspondences in the volume adds to the computational requirement while not facilitating convergence to the minima of the alignment criterion (Akselrod-Ballin et al., 2011; Clatz et al., 2005; Hedouin et al., 2017). We have exploited a patch selection scheme to select patches that contain pixels from regions with rich features that help improve the matching process. In this scheme, patches are selected based on their pixel variance; i.e., only patches with top 60% pixel variance are incorporated into the matching criterion. Eq. (2), therefore, is rewritten as:

(3) $$f\left(I,I^{\prime }\right)=\sum_{k\in S_{v}}{\left\|p_{k}(I)-p_{k}\left(I^{\prime }\right)\right\|}_{2}^{2},$$

where the set $S_{v}\subset S$ indexes the patches from the input slice *I* with the top 60% greatest pixel variances. Therefore, this patch selection criterion identified patches that drive the alignment and reduced the computational cost of the matching criterion by 40%.

#### 2.1.3. EPI fMRI acquisition

The ease with which the correct alignment of a slice with a reference volume can be computed depends on the amount of brain tissue visible in the slice. Slices at the very top and bottom of the brain may have good matches in several positions due to the reduced amount of brain tissue visible in those slices, whereas slices in the middle of the brain more frequently have a unique good match. Consequently, it can be advantageous to consider more than one slice at a time in computing the alignment. Simultaneous multi-slice (SMS) acquisitions reconstruct two or more maximally separated slices at the same time and are a good choice for alignment. Slices may be acquired in an interleaved order that may be used to improve alignment by computing the alignment using two or more slices that are consecutively acquired in time, rather than using slices that are physically adjacent to each other. Limited features in border slices may lead to misalignment and errors in SVR. As a result, correct alignment of a single slice is more challenging than alignment of two or more slices that cover the anatomy at different locations. EPI slices in fMRI are typically acquired in an interleaved manner, primarily to avoid cross-talk between slices, with the number of slices skipped referred to as the interleaving parameter (Parker et al., 2017). This strengthens feature matching in SVR; and makes SVR robust especially at border slices.

In an SMS acquisition scheme, patches taken from *N* simultaneously acquired slices sample the anatomy at positions that are at $\frac{1}{N}$ distances of each other, where FOV_*s*_ is the field-of-view in the slice select direction (i.e., the number of slices times slice thickness). The *N* slices that are acquired at the same time, have the same motion state; therefore they can regularize SVR and improve its accuracy (Marami et al., 2019). Our simultaneous-*N*-slice-based similarity minimization is thus written as

(4) $$\underset{\theta }{\text{min}}\sum_{i=1}^{N}\sum_{k\in S_{v}^{i}}\tau_{i}{\left\|p_{k}\left(I_{i}\right)-p_{k}\left(T_{n_{i}}(J;\theta )\right)\right\|}_{2}^{2},$$

where the subscript *n*_*i*_ denotes the index of the *i*th slice from the volume, and ${\left\{S_{v}^{i}\right\}}_{i=1:N}$ indexes patches that are selected from the *i*-th input slice. A linear interpolator is employed in the transformer *T*_*n*_(·, ·) to make a trade-off between accuracy and computational efficiency. *N* weight parameters *τ*_1:*N*_ are used to balance between the *N* similarity terms related to the *N* simultaneous slices. As a result, more flexible strategies can be applied to fine tune the stability and accuracy of the registration by setting different values of *τ*_*i*_ for different input slices. For example, the value of *τ*_*i*_ can be chosen dynamically based on the area covered by the slice *I*_*i*_, approximated by pixel intensities; i.e., if a slice contains more features, *τ*_*i*_ is set to a larger value:

(5) $$\tau_{i}=\frac{\tau_{i}^{\prime }}{\sum_{i}\tau_{i}^{\prime }},\;\tau_{i}^{\prime }=\text{exp}\left(\frac{1}{\sigma }\sum_{k}\left|I_{i}(k)\right|\right),$$

where *σ* > 0 is a scale parameter for the exponential function. The *τ*_*i*_ value could also be set manually according to empirical knowledge. In the particular case of single slice acquisition, when *τ*_2:*N*_ = 0, Eq. (4) leads to the ordinary SVR problem.

#### 2.1.4. Theory of our algorithm

Eq. (4) can be solved by either a single-valued optimizer, e.g., the Powell algorithm (Powell, 1964), or a multi-valued optimizer such as the Levenberg–Marquardt (LM) algorithm (Levenberg, 1944; Marquardt, 1963). We chose the latter for its faster convergence. Eq. (4) poses a standard nonlinear unconstrained least squares problem with respect to the motion states ***θ***, where the set of $\sum_{i=1}^{N}\left|S_{v}^{i}\right|$ datum pairs is described as

(6) $$\overset{N}{\underset{i=1}{\cup }}\left\{\left(f_{k}^{i}(\theta ),0\right)|k\in S_{v}^{i}\right\},$$

where $f_{k}^{i}(\theta )$ is written as

(7) $$f_{k}^{i}(\theta )=\tau_{i}{\left\|p_{k}\left(I_{i}\right)-p_{k}\left(T_{n_{i}}(J;\theta )\right)\right\|}_{2}^{2}.$$

The LM algorithm involves iterations of this form:

(8) $$\theta^{t+1}=\theta^{t}+\delta^{t},$$

where ***δ***^*t*^ indicates the search direction and the step size that can maximally reduce the objective function (i.e., the similarity function in Eq. (4)) at the *t*th iteration.

According to the first-order Taylor series expansion, the similarity at the (*t* + 1)th iteration is approximated by

(9) $$f_{k}^{i}\left(\theta^{t+1}\right)=f_{k}^{i}\left(\theta^{t}+\delta^{t}\right)\approx f_{k}^{i}\left(\theta^{t}\right)+\frac{\partial f_{k}^{i}\left(\theta^{t}\right)}{\partial \theta^{t}}\delta_{k}^{t}.$$

It is derived in the LM algorithm that ***δ***^*t*^ can be solved analytically from the following closed-form solution

(10) $$\delta^{t}={\left(J^{T}J+\lambda \text{diag}\left(J^{T}J\right)\right)}^{-1}J^{T}f\left(\theta^{t}\right),$$

where all the $f_{k}^{i}\left(\theta^{t}\right)$ values with respect to ***θ***^*t*^ are combined into a vector **f**(***θ***^*t*^); $J\in {\mathbb{R}}^{M\times 6}$ denotes the Jacobian matrix of **f**(***θ***^*t*^) for $M=\sum_{i=1}^{N}\left|S_{v}^{i}\right|$; diag(**J**^*T*^**J**) denotes the diagonal matrix consisting of the diagonal elements of **J**^*T*^**J**; and *λ* is a weight parameter.

The iterative LM algorithm requires an initial guess for the solution, i.e., ***θ***^0^, which is critical to the final solution. We initialize the solution ***θ***^0^ for slice *I*_*i*_ by the motion state of the slice acquired prior to it. The algorithm stops if any of the following criteria is reached: 1) both the actual and predicted relative reductions in the sum of squares are at most 10^−8^; 2) the relative error between two consecutive iterations is at most 10^−8^; and 3) the cosine of the angle between the functions evaluated at ***θ***^*t*^ and any column of the Jacobian is at most 10^−5^ in absolute value.

The solution to *dnew*^*t*^ shown in Eq. (10) involves a Jacobian matrix, matrix multiplications, and a matrix inversion. Although the matrix inversion has a cubic order $\left(O\left(n^{3}\right)\right)$ of computational complexity, the matrix being inverted is of size 6 × 6. The matrix multiplications can be performed efficiently by applying a QR decomposition on the Jacobian matrix **J**. The Jacobian matrix has *M* × 6 elements to be computed. The computational cost of computing **J** increases linearly with a factor 6 with the number of patches in Eq. (4). As a result, computing the Jacobian matrix **J** is the main computational burden in Eq. (10), in particular over a large number of empirical datum pairs such as image data. Based on our results on real data, the LM algorithm takes 60% execution time in computing the Jacobian matrix, 21% for QR decomposition, and 19% for the remaining steps. Thus, here we focus on speeding up the Jacobian matrix computation for the LM algorithm.

We use a finite difference method to compute the Jacobian matrix. The forward difference for the Jacobian is written as

(11) $$J={\left[J^{1},\;\ldots ,\;J^{N}\right]}^{T},$$

where the sub-matrices are found by

(12) $$J_{kj}^{i}=\frac{1}{h_{j}}\left(f_{k}^{i}\left(\theta_{j}+h_{j}\right)-f_{k}^{i}\left(\theta_{j}\right)\right),$$

with *i* = 1, 2, … *, N, j* = 1, 2, … 6, and *h*_*j*_ > 0 being the step length of the forward difference obtained from

(13) $$h_{j}=\text{max}\left(\sqrt{\text{max}\left(10^{-11},\epsilon \right)},\sqrt{\text{max}\left(10^{-11},\epsilon \right)}\cdot \left|\theta_{j}\right|\right),$$

where *ϵ* denotes the machine precision.

**Fig. 1.**
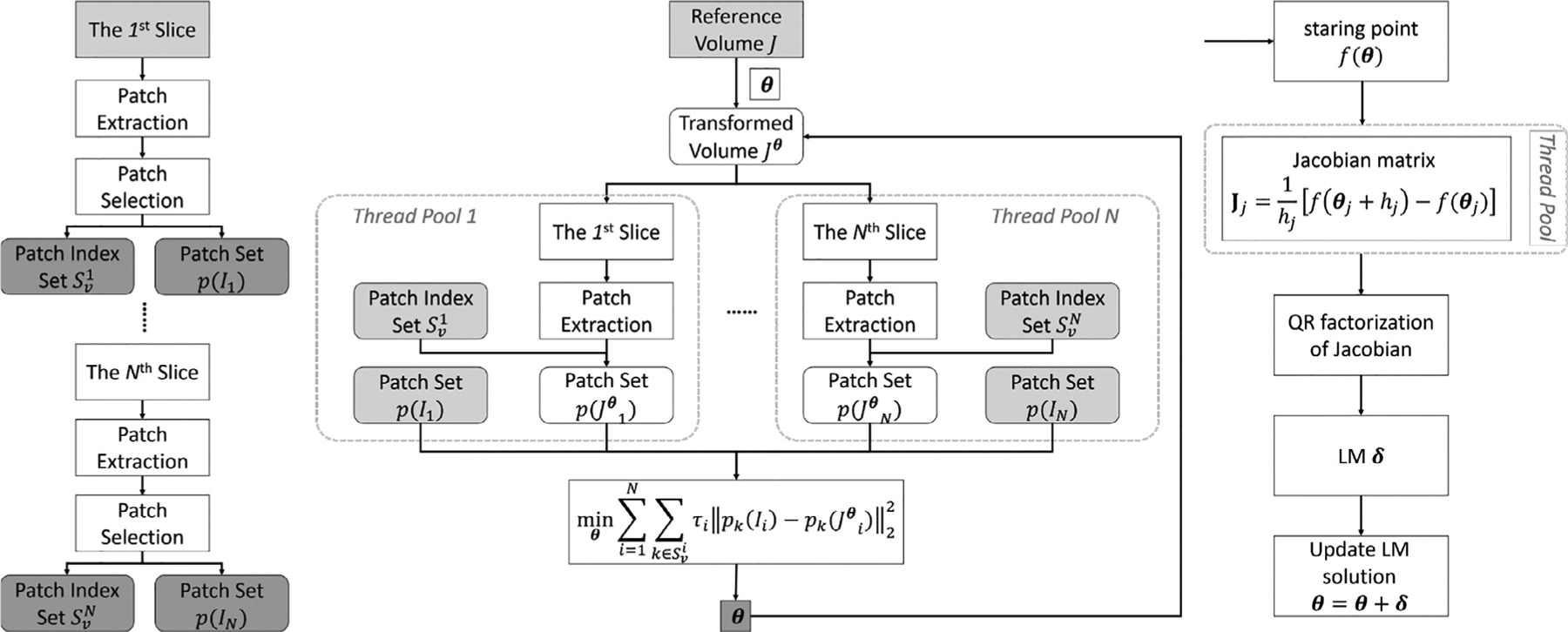
The flow diagram of the proposed slice-to-volume registration algorithm. The inputs and the outputs are highlighted by the light and dark gray boxes respectively. (left) The patch extraction and selection processes. (middle) The slice-to-volume registration process. (right) Iterative optimization.

The *N* sub-matrices **J**^*i*^ can be computed independently over the simultaneous-*N*-slice by using *N* sets of threads, leading to *N* times acceleration. For each sub-matrix **J**^*i*^, the function values $f_{k}^{i}\left(\theta_{j}+h_{j}\right)$ only depend on *θ*_*j*_. Hence, another 6 threads are employed to compute the 6 columns of **J**^*i*^ in parallel, resulting in an additional 6 times acceleration for the Jacobian matrix computation. The parallelization and the flowchart of the complete SVR algorithm are depicted in Fig. 1.

In summary, our SVR algorithm is accelerated for real-time performance by optimizing the matching criterion and parallelizing the optimization algorithm. The patch-based matching criterion leads to fewer iterations to enable the optimization algorithm to rapidly converge. The patch selection scheme identifies 60% of patches for the similarity evaluations, which constitutes a saving of 40% of computational costs. Further, the multi-threaded LM algorithm speeds up the Jacobian matrix computation by 6*N* × by the parallelization design. We will show the actual acceleration performance in the results section.

#### 2.1.5. Auto-calibration for reference volume

When our motion monitoring system is launched with an fMRI scan, it starts with an auto-calibration stage that uses our real-time SVR algorithm to find a reference volume. The auto-calibration stage works as follows: the first fMRI volume is regarded as a provisional reference volume. The slices of the second volume are registered to this volume using SVR as they are acquired. If the motion measurements on all slices of the second volume are below a predefined threshold, the first volume is confirmed as the reference volume as this indicates no motion was detected within any of the slices of the first and second volume. If the motion measurements do not pass the threshold condition, the first volume is discarded, the second volume is regarded as the provisional reference, and all slices of the third volume are registered to the second volume. The motion measurements between the second and third volumes are then evaluated and compared against the threshold. This process continues until no motion is detected within the slices of two consecutive volumes, which means that the first volume of the two is chosen and used as the motion-free reference for SVR.

#### 2.1.6. Motion monitoring system

**Fig. 2.**
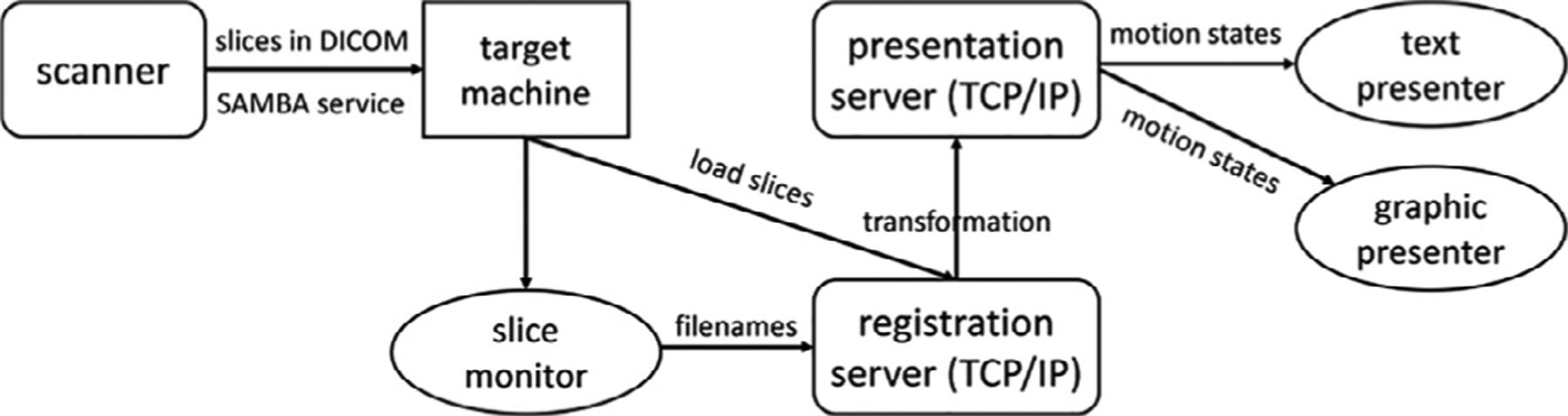
Architecture of our real-time motion monitoring system (SLIMM). Slices are transferred through SAMBA from the scanner computer to a workstation that runs our algorithm. The slice monitoring module watches the new slice acquisitions and sends the file names of the new slices to the registration server. Motion measurements are conducted by the registration service. The measured transformations (each contains 6 motion parameters) are dispatched to the presentation server and shown by one or more client services.

Our fMRI sequence writes the slices as they are acquired, in DICOM format, to a local file system or a network mapped file system mounted using the SMB protocol on the MRI scanner. We export a file system from a Linux workstation to the scanner, and execute the SVR alignment on the workstation. The motion measurements are then dispatched asyn-chronously to one or more receiving clients. The clients may present the motion trajectory through a graphic display, or as a text stream, or may be used to modify the visual or audio stimulus presented to the subject. The architecture of our motion monitoring system is shown in Fig. 2.

### 2.2. Material

To assess our method (SLIMM), we acquired extensive real fMRI data with real, in-scanner motion. All the prospective imaging experiments for this study were done on 3T Siemens MR scanners (Siemens Healthcare, Erlangen, Germany). All scans were performed in accordance with a protocol approved by the institutional review board committee. Some important parameters of the fMRI sequences used in this study are shown in Table 1, and the data sets are described below. Beside that, all scans used the parameters: pixel bandwidth of 2230Hz/pixel, field of view of 192 mm × 192 mm × 108 mm, in-plane acceleration factor of 2, and flip angle of 90 degrees. Conventional fMRI sequences of ascending order use the slice order of [1: 1: *n*] in the directions from foot to head with *n* being the number of slices per volume, and *i*: *j*: *k* denoting a number sequence from *i* to *k* by a step *j*. Our fMRI acquisitions used the interleaved scheme, where an even-first ascending order was incorporated. An interleave factor of *n*_*i*_ in our fMRI sequences means that the slice order is [2 : *n*_*i*_ : *n*, 1 : *n*_*i*_ : *n* − 1]. Moreover, SMS was accomplished in combination of the interleaved scheme. An SMS factor of *n*_*SMS*_ in our fMRI acquisitions means that *n*_*SMS*_ slices are simultaneously acquired and the slice order of these *n*_*SMS*_ slices is $\left(i,i+j_{1},\ldots ,i+j_{n_{SMS}-1}\right)$ with $j_{k}=k\cdot \frac{n}{n_{SMS}}$ and *i* ∈ {1, …, *n*}. For example, with an SMS factor of 2, an interleave factor of 2, and number of slices *n* = 36, the slice order is (2, 20), (4, 22), (6, 24), …, (18, 36), (1, 19), (3, 21), (5, 23), …, (17, 35), where (*i*_1_, *i*_2_) indicates slices *i*_1_ and *i*_2_ are simultaneously acquired.

#### Electromagnetic Sensor Motion-Tracking Data Set (EM-Tracking).

To assess the accuracy of our approach in motion measurement with real in-scanner motion, it is desirable to construct gold standard motion measurements as the reference motion points, and also to acquire the motion-free reference data. We conducted fMRI scans in 6 volunteer subjects with real, in-scanner motion. For each volunteer, two fMRI time series were acquired: in one scan the volunteer stayed still and the acquired scan was used as the “no motion ” reference. During the other scan the volunteer was instructed to move via audio cues. We used an electromagnetic (EM) motion tracking sensor (Afacan et al., 2019) developed by Robin Medical Inc. (Baltimore, MD) to monitor motion during scans. Motion measurements from the EM tracker were used as the reference.

#### Optical Motion Tracking Data Set.

To thoroughly assess the accuracy of our approach in motion measurement, gold standard motion measurements for real in-scanner motion from various motion sensors are required. As a result, we employed an optical motion tracking system to establish the gold standard measurements. For one volunteer subject, we recorded two head motion tracking data sequences using the Kineticor camera system (Siemens and KinetiCor, 2018) (KinetiCor Inc., Honolulu, Hawaii) during fMRI scans in which the volunteer performed real, in-scanner motion. During the scan, the volunteer was instructed to perform nodding head motion. The measurements obtained from the Kineticor optical motion tracking system were used as reference motion points for this data set.

#### Patient Data Set.

Beyond the volunteers’ scans where the volunteers were instructed to move, it is desirable to assess our approach in the real, unconstrained acquisitions. Therefore, we acquired this data set from 3 patients with real, in-scanner motion, containing 2 resting-state fMRI scans and a task-based fMRI scan (finger tapping). For these scans the patients were encouraged to stay still, but they moved.

#### Healthy Brain Network (HBN) Data Set.

To evaluate the efficacy of our auto-calibration module on large real pediatric data, we used the HBN data set (Alexander *et al.*, 2017), which contains resting state fMRI scans of 251 subjects. The age range of these subjects is from 5.8 to 21.4 years. The fMRI time series have 375 measurements, slice thickness of 2.4 mm, number of slices = 60, and matrix size = 84 × 84.

### 2.3. Experimental plan

We implemented our algorithms in C++. All experiments reported in this work were conducted on a workstation with 20 cores of Intel(R) Xeon(R) CPU E5–2698 v4 @ 2.20 GHz.

Since VVR methods have been popular and used in the past, we implemented a VVR method to compare to. The VVR method, that we called the VVR-LM in this work, was implemented with a mean squares metric obtained from Numpy, a linear interpolator provided by SimpleITK for resampling image, and an LM optimizer implemented by Scipy. The rigid transform was implemented by 3 Euler angles and 3 translations, and initialized by the transform estimate of the prior volume. VVR-LM is able to perform in real-time, and thus it enables motion monitoring. As a result, it was designed with different aims from those retrospective VVR methods, such as SPM (Penny et al., 2007) and FSL/FLIRT (Jenkinson et al., 2002; Jenkinson and Smith, 2001). VVR-LM we implemented in this work aims at measuring motion in real-time, while those retrospective VVR methods target more complicated registration designs, e.g., performing both intra- and inter-modality registrations, and removing motion artifacts from the images.

VVR-LM was used to compare the performance of using the SVR strategies in comparison to using the VVR strategy. Our SVR method described in Section 2.1 above was called SLIMM in the experiments.

The assessments of our approach focused on the accuracy and actual speed of motion measurement, and acquisition efficiency and quality. We did not compare to alternative approaches that were too slow to complete the motion monitoring before the next slice was acquired.

#### 2.3.1. Accuracy of motion measurement

In order to accurately detect the volumes during which motion has occurred, it is important that the motion measurement be accurate. In order to accurately identify sections of data that are corrupted by motion, for which additional data will be acquired, accurate estimation of the motion of each slice is necessary. In addition, the motion measurement should not wrongly indicate that there is motion when the subject is holding still. We thus conducted extensive experiments to assess the accuracy of our motion measurement.

We assess the accuracy of motion measurement through two types of criteria:

- Motion measurement error. We calculated and reported the error in terms of both motion transformation parameters and slice displacement (SD) on the data sets where reference motion parameters are available.
- tSNR from the retrospective correction. Temporal signal-to-noise ratio (tSNR) is an important metric to assess the fMRI data quality (Murphy et al., 2007). The calculation of tSNR is required to run on the motion-corrected data. Therefore, the accuracy of our motion measurement is highest when the tSNR is highest, because the tSNR is reduced by increased signal variance when the alignment is wrong.

##### Accuracy through Motion Measurement Error.

We had two real motion data sets acquired along with gold standard motion measurements. The errors in motion parameters and SD can be investigated on these data sets to assess the accuracy of our approach in motion measurement by referring to the gold standard motion measurements. The motion parameter error **e**_*p*_(*k*) at the *k*th slice in an fMRI data sequence was obtained from

(14) $$e_{p}(k)=\left|p_{ref}(k)-p_{m}(k)\right|,$$

where **p**_*ref*_ and **p**_*m*_ respectively denote the motion transformation parameters, consisting of 3 rotational and 3 translational parameters, obtained from the reference and the image alignment-based motion measurement method (SLIMM or VVR-LM in the experiments), and |·| was applied for elementwise absolute value.

Similarly, the SD error *e*_*d*_(*k*) at the *k*th slice in an fMRI data sequence was computed from

(15) $$e_{d}(k)=\left|d_{ref}(k)-d_{m}(k)\right|,$$

where *d*_*ref*_ and *d*_*m*_ respectively denote the SD obtained from the reference and the motion measurement method by using Power et al. (2012) approach that is used to compute frame displacement.

Frame displacement (FD) assessment was first proposed by Power et al. (2012) in order to characterize the amount of head motion, and FD was used to demonstrate that widely used retrospective motion correction strategies do not eliminate the influence of motion on the BOLD signal. In that paper, each volume *i* of an fMRI time series was aligned to a reference volume, providing a rigid body transformation **T**_*i*_. FD was defined as: $FD_{i}=\left|\Delta \alpha^{i}\right|+\left|\Delta \beta^{i}\right|+\left|\Delta \gamma^{i}\right|+\left|\Delta t_{x}^{i}\right|+\left|\Delta t_{y}^{i}\right|+\left|\Delta t_{z}^{i}\right|,$, where $\Delta t_{x}^{i}=t_{x}^{\left(i-1\right)}-t_{x}^{i}$, and similarly for the other rigid body parameters $\left[\alpha^{i},\beta^{i},\gamma^{i},t_{x}^{i},t_{y}^{i},t_{z}^{i}\right]$. Rotation parameters were converted from degrees to millimeters by computing the arc length subtended by the angle on the surface of a sphere of radius 50 mm. This radius is approximately the average distance from the center of the head to the cortex. Different numerical values could be used for different sized heads.

The FD can be generalized to slices by considering the possibility that each slice, not just each volume, may undergo an independent rigid body transformation. In this case, for a slice *j* of volume *i*, the SD can be denoted: $SD_{ji}=\left|\Delta \alpha^{ji}\right|+\left|\Delta \beta^{ji}\right|+\left|\Delta \gamma^{ji}\right|+\left|\Delta t_{x}^{ji}\right|+\left|\Delta t_{y}^{ji}\right|+\left|\Delta t_{z}^{ji}\right|$, where the differences in displacement are computed similarly to that of the volumes.

As presented in Section 2.2, the reference motion parameters (gold standard measurements) were available on the EM-Tracking and the optical motion tracking data sets. We ran SLIMM and VVR-LM separately to measure the motion at the slice and the volume level, respectively, on the two data sets. We were thus able to evaluate the motion parameter error and the SD error. Since VVR-LM measured the motion at the volume level, we generated the motion measurements at the slice level for VVR-LM by assigning the measurements of each volume to its all slices. Our goal in this experiment was to validate SLIMM performed with smaller errors than VVR-LM in terms of both above criteria, indicating SLIMM performed more accurately than VVR-LM in motion measurement.

##### Accuracy through retrospective correction.

As addressed above, tSNR is an important metric to assess the fMRI data quality (Murphy et al., 2007). However, the calculation of tSNR was required to run on the motion-corrected data. We thus corrected the volumes by reconstructing volumes from individually registered slices. For this, we used a slice acquisition model, as a slice-based motion correction technique, to reconstruct time series of fMRI volumes from motion-corrected slices. Let **x** be the vector form of the reconstructed volume, and **y**_*i*_ the vector form of the *i*-th acquired slice *I*_*i*_. The slice acquisition model is written as

(16) $$y_{i}=P_{i}S_{i}T_{i}x+\mu_{i},$$

where **T**_*i*_ transforms **x** according to ***θ***_*i*_ (**T**_*i*_ defines the inverse transform according to ***θ***_*i*_); **S**_*i*_ denotes the slice profile which is approximated here by a truncated Gaussian for the 2D gradient-echo EPI for fMRI; **P**_*i*_ extracts the *i*th slice from the volume; and ***μ***_*i*_ denotes an additive noise term. Assuming that the noise ***μ***_*i*_ yields a zero-mean Gaussian distribution, the motion-free volume **x** can be reconstructed from

(17) $$\underset{x}{\text{min}}\sum_{i}{\left\|y_{i}-P_{i}S_{i}T_{i}x\right\|}_{2}^{2}+\lambda ∥\nabla x{∥}_{1},$$

The first term is least squares and the second term is total variation regularization weighted by *λ >* 0; where ∇ denotes the derivative operation. We solve Eq. (17) by gradient descent, where the update for **x** at the *k*th iteration is found by

(18) $$x^{\left(k\right)}=x^{\left(k-1\right)}-\eta \left(g\left(x^{\left(k-1\right)}\right)+\lambda r\left(x^{\left(k-1\right)}\right)\right),$$

with the derivatives of the data fidelity term *g*(·) and the regularization term *r*(·) defined by

$$g(x)=\sum_{i}T_{i}^{T}S_{i}^{T}P_{i}^{T}\left(P_{i}S_{i}T_{i}x-y_{i}\right),$$

(19) $$r(x)=\left(I-\nabla^{-1}\right)\text{sign}(\nabla x).$$

where $T_{i}^{T}$ denotes the inverse transform of *T*_*i*_ (corresponding to ***θ***_*i*_); $S_{i}^{T}$ denotes the convolution kernel with flip of **S**_*i*_ from left to right, top to bottom; $P_{i}^{T}$ performs zero-slice padding for the *i*th slice; **I** denotes the identity matrix; ∇^−1^ computes the backward derivative; and the operator *sign*(·) computes the sign of its input. In Eq. (18), we set the learning rate *η* = 0.1, and the regularization weight *λ* = 10^−3^. Note that the slices **y**_*i*_ may move out-of-plane after performing the SVR, leading to artifacts in reconstruction. Total variation regularization, therefore, enhances the reconstruction by edge-preserving smoothing.

We analyzed the performance gained from our SLIMM approach in terms of tSNR. In the experiments, we retrospectively corrected the data with motion at the slice and volume level, through the motion measurements obtained from SLIMM and VVR-LM, respectively. We were then able to compute the tSNR over these motion-corrected data for the comparisons. In addition, on the EM-Tracking data set, the reference scans containing the data with no motion (from the “no motion ” reference scans during which the volunteers were asked to hold still) were available and adopted as the references for the comparisons in tSNR. In those comparisons, frame censoring was also incorporated to investigate the tSNRs and their respective changes. When frame censoring was turned off, all 96 corrected volumes of each subject were used to compute the tSNRs. When frame censoring was enabled, we excluded motion-corrupted volumes and computed tSNRs over the remaining corrected volumes. We used a method similar to Power et al. (2012) to identify motion-corrupted volumes, where motion measurements of each slice were compared against a threshold (1/4th of slice thickness). We removed a volume if it had ≥ 1 motion-corrupted slices.

#### 2.3.2. Acquisition efficiency and quality

One goal of our motion monitoring approach, SLIMM, is to reduce the scan duration and to improve the data quality through the online frame censoring and real-time feedback to the subject as well as the scanner operator. The online frame censoring enabled adjusting the length of acquisition dynamically and adaptively. It collected the motion-free volumes only, which were identified through the real-time slice-by-slice motion measurements, and automatically increased the length of acquisition until sufficient motion-free volumes have been collected. SLIMM also provided real-time feedback during the scan, which has been demonstrated to be effective to reduce scan duration (Dosenbach et al., 2017; Greene et al., 2018).

##### Motion Identification.

Both the online frame censoring and the real-time feedback relies on the motion identification incorporated in the motion monitoring system. In SLIMM, we identified the motion from the motion measurements by thresholding the SD according to a predefined motion threshold parameter. If a slice was measured to displace over the motion threshold, then it was identified as a motion-corrupted slice. If a volume contained any motion-corrupted slices, then this volume was identified as a motion-corrupted volume, and was excluded from the data collection with the online frame censoring protocol.

As presented above, the motion threshold is critical to motion identification. We thus investigated appropriate threshold values for SLIMM. In the VVR-based method, the motion threshold from the range of [0.2,0.6] is widely used on frame displacements (FD) that are computed volume by volume, from high to low. In our assessments of SLIMM, the motion threshold was imposed on SDs computed slice by slice. Therefore, we first verified if or not the motion identified through the SDs was consistent with the FDs for the same volumes, i.e., were all the SDs of those volumes less than *t*_*FD*_ as well when the FDs of those volumes were less than *t*_*FD*_, for a motion threshold *t*_*FD*_ ∈ [0.2, 0.6]? In this experiment, on the EM-Tracking data set where the gold standard motion measurements were available, we collected the volumes with the FDs *< t*_*FD*_ according to the measurements of VVR-LM. We calculated the SDs of the same volumes over the gold standard motion measurements, and then constructed distributions of these SDs to analyze the results. If the SDs were consistent with the FDs, we could directly use the same threshold values in [0.2,0.6] for SLIMM; otherwise, we had to find the appropriate threshold values corresponding to those on the FDs in this range.

In the latter case (not consistent), we first computed the FD measured by using VVR-LM, and found out all the fMRI data volumes of FD ≥ *t*_*FD*_, as the motion-corrupted volumes, for a motion threshold *t*_*FD*_ ∈ [0.2, 0.6], on the EM-Tracking data set where the gold standard motion measurements were available. Since VVR-LM may identify by mistake these motion-corrupted volumes from the less accurate FDs, we excluded 20% of these volumes that led to the top 20% largest differences in SD between the measurements of gold standard and VVR-LM, to form a set of motion-corrupted volumes.

SLIMM should identify all the volumes in this set as motion-corrupted. To this end, we looked for a motion threshold *t* that should be less than or equal to all the maximum SDs of each volume in this set, to ensure that at least one slice of each volume was motion-corrupted. The threshold *t* was thus found by

(20) $$t=\text{min}{\left\{\text{max}{\left\{D\right\}}_{j}\right\}}_{j=1}^{N},$$

where {*D*}_*j*_ denotes the set of SDs obtained from all slices of the *j*-th volume, for *j* = 1, 2, … *, N* motion-corrupted volumes identified using the above-mentioned method. We thus found the corresponding motion thresholds on SD to those of FD.

##### Online Frame Censoring.

We evaluated the acquisition lengths, that were adaptively determined by SLIMM and VVR-LM, for collecting a desired number of motion-free volumes. More importantly, the quality of the collected data was assessed in terms of tSNR, to demonstrate that the motion monitoring system made a correct decision on increasing the length of the acquisition (i.e., with online frame censoring the motion-corrupted volumes were successfully excluded from the data collection). In the experiments, we set the desired number of volumes to be 80% of the total number of volumes on each fMRI data sequence of all data sets that we acquired in this work, and the rest of the 20% of volumes were viewed as the over-scanned volumes in the face of unknown motion-induced data loss without any motion monitoring system applied, as done in the retrospective frame censoring-based methods. It indicated by this setting that the acquisition lengths were manually increased by a fixed rate of 25% (20%÷80%) with no motion monitoring. In the experiments, we ran SLIMM and VVR-LM separately on each of the data sequences according to the above online frame censoring protocol. We expected to achieve increased acquisition lengths of less than 25% and higher tSNRs with motion monitoring, to demonstrate that motion monitoring can reduce the acquisition duration and improve the data quality.

##### Online Feedback.

We also ran an experiment to validate that SLIMM was able to successfully suggest the scanner operator to intervene in the acquisition through the real-time feedback, if the continuous motion was observed. In the experiment, the motion monitoring system would suggest the operator to stop the acquisition until the subject stopped moving, if no motion-free volumes were collected in the past 30 seconds. During the acquisition, we instructed the volunteer to move continuously, as described in Section 2.2, optical motion tracking data set, to trigger the suggestion of the motion monitoring system. We compared SLIMM to VVR-LM in time when the suggestion for intervention was triggered, since earlier intervention indicated more reduction in the scan duration.

#### 2.3.3. Efficacy of auto-calibration for reference volume

The reference volume is important in SVR, since it is damaging to the SVR if the reference volume contains any motion. Therefore, for a comprehensive investigation, we examined the practical efficacy of our auto-calibration method for the reference volume on the HBN data set (a large scale data set containing the fMRI scans of 251 pediatric subjects).

#### 2.3.4. Computational evaluation

The real-time motion measurement in our approach means that the motion of a slice can be measured within the period of its next consecutive slice acquisition. We used the slices processed per second (SPS) as an indicator to assess the speed of motion measurement. We evaluated the SPS for SLIMM on the slices from all data sets that we acquired.

## 3. Results

### 3.1. Accuracy of motion measurement

#### 3.1.1. Accuracy through motion measurement error

Table 2 shows the mean and standard deviation of motion measurement errors obtained from VVR-LM and SLIMM on the EM-Tracking data set. Our approach SLIMM outperformed VVR-LM. We also performed *t*-test on the errors of motion parameters. In the test, we assumed the errors of the motion parameters obtained from the two methods came from normal distributions with unknown, but equal, variances. At 5% threshold for the significance level, the hypothesis was rejected as the *p*-values were 3.7 ×10^−15^ for the translation errors and 5.4 ×10^−25^ for the rotation errors, respectively. This indicated that the transformation errors of the two methods statistically yielded different distributions, and the difference was significant. Consequently, the difference showed the average accuracy gained from slice-by-slice motion measurement in our SVR method in comparison to the method relying on volume-by-volume motion measurement.

**Fig. 3.**
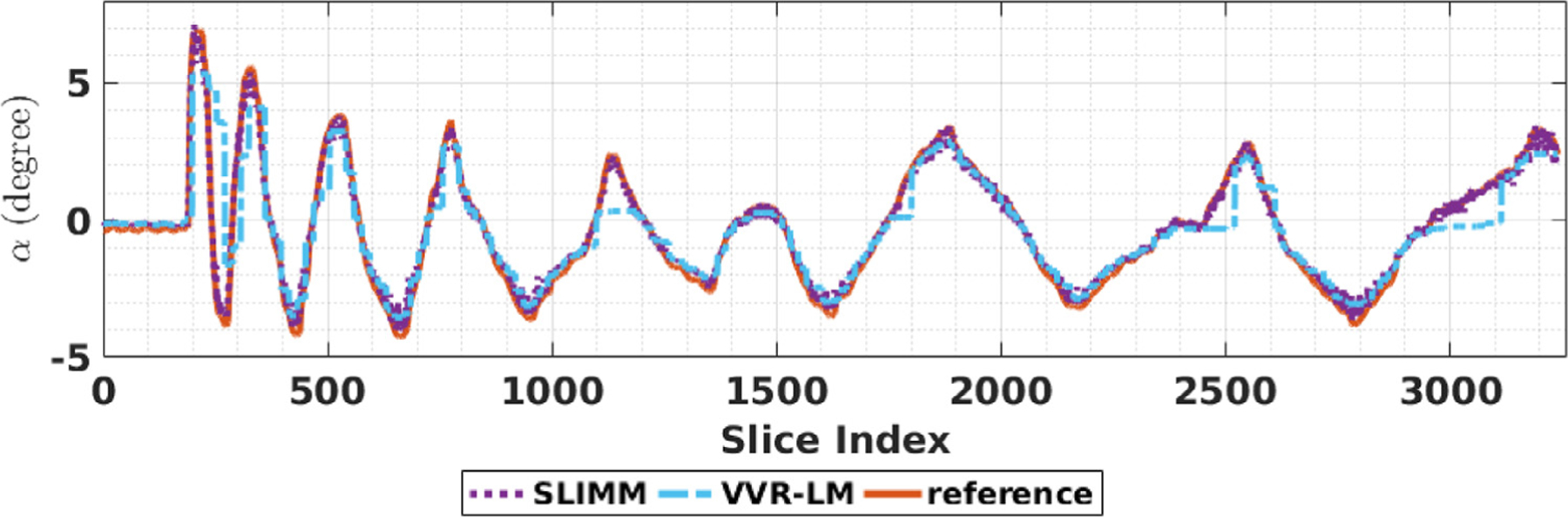
Motion measurements in terms of rotational motion parameters *α* on the optical motion tracking data set. Our method, SLIMM closely followed the real, reference motion pattern measured by the Kineticor optical motion tracking system. The magnitudes of total differences of the measurements between the reference and SLIMM/VVR-LM in *α* were SLIMM = 726.4, VVR-LM = 1740.0.

Fig. 3 shows the motion measurements in terms of rotation parameters *α* obtained from SLIMM, VVR-LM, and the optical motion tracking system (camera) on the optical motion tracking data set. It can be seen that both VVR-LM and our method, SLIMM, closely followed the real, reference motion pattern measured by the optical tracker. As shown in Table 3, the overall mean and standard deviation of the motion measurement errors in terms of SD obtained from SLIMM are lower than those obtained from VVR-LM. As the subject moved faster at the beginning compared to the end of the sequence, we separately analyzed the data points from the range of [200, 300]. The means and standard deviations of the errors in SD obtained from VVR-LM and SLIMM were shown in Table 3. Our method outperformed VVR-LM in this period of fast motion.

Overall, our SLIMM approach considerably outperformed the baseline VVR-LM consistently on the real motion data sets in terms of motion measurement error.

#### 3.1.2. Accuracy through retrospective correction

**Fig. 4.**
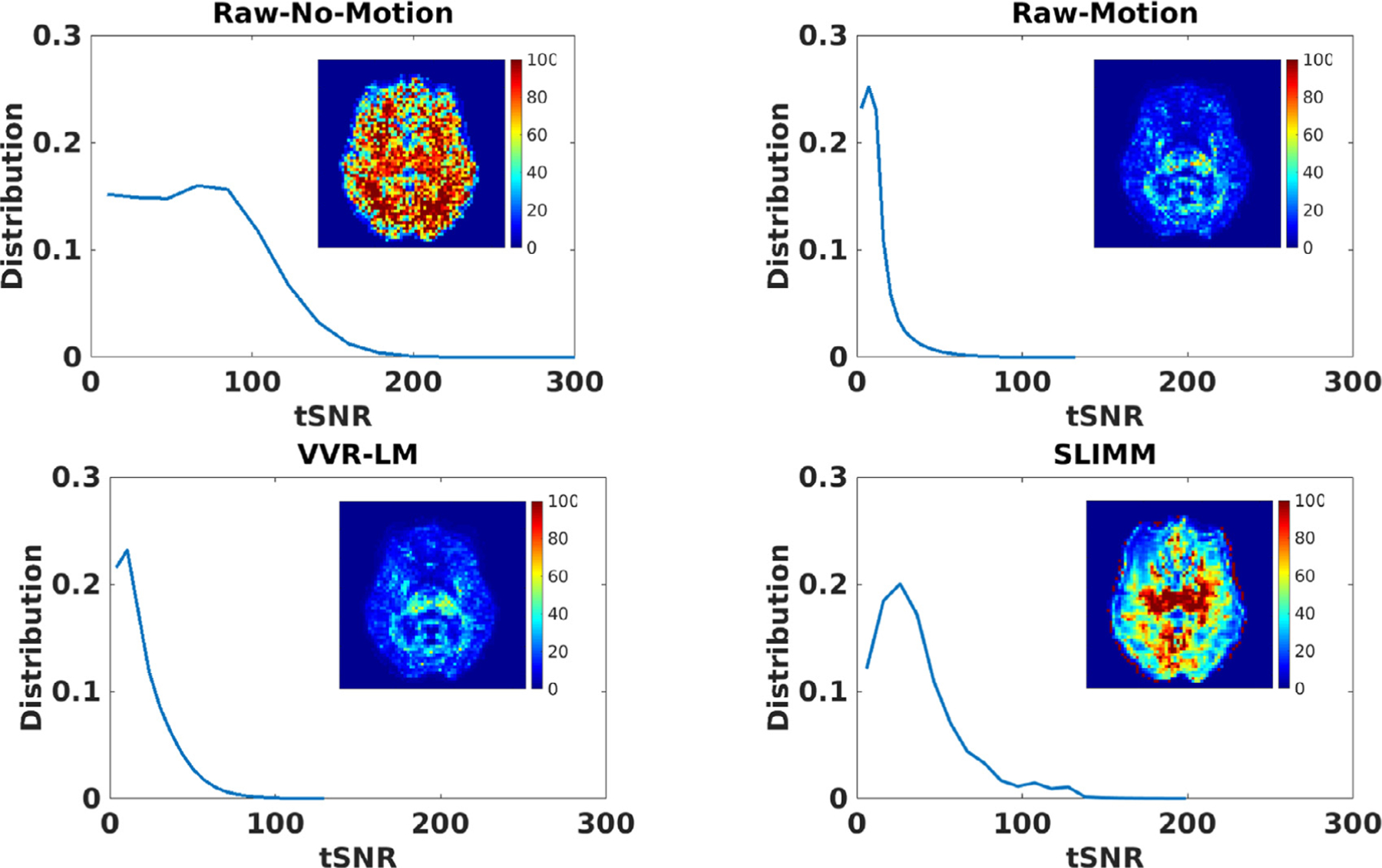
Distributions of the tSNRs over data from all subjects calculated by four methods compared to Raw-No-Motion and Raw-Motion data acquired with no motion and in-scanner motion respectively. The tSNR map calculated from the reconstructed fMRI time series by each method is also shown for an axial slice of a representative subject in each subfigure. These results show that our SVR method substantially improved the tSNR of the Raw-Motion data and outperformed VVR-LM. While tSNR is lost naturally due to motion in fMRI, the SVR methods were able to recover a large portion of tSNR despite the continuous subject movements that occurred during these scans. Our method, SLIMM, generated the best results according to the average tSNR values reported in Table 4.

Fig. 4 shows the distributions of the tSNR scores obtained from the two motion measurement methods on the EM-Tracking data set of all 6 subjects. We denoted by Raw-No-Motion the data from the “no motion” reference scans during which the volunteers were asked to hold still, and by Raw-Motion the data with motion. The numbers of the remaining volumes of the 6 subjects were 41, 79, 68, 48, 62, and 54, respectively, when applied frame censoring. Table 4 shows the average tSNR scores over all the voxels of the tSNR volumes on the EM-Tracking data set of the 6 subjects with the frame censoring turned on and off. We see that the tSNRs of Raw-No-Motion had very slight changes when the frame censoring was on and off, whereas, both SLIMM and VVR-LM improved the tSNRs, as compared to the Raw-Motion. Our method, SLIMM, considerably outperformed VVR-LM. A tSNR map from a representative subject is also shown in Fig. 4, from which we can see results that are consistent with the histograms and the average tSNRs.

Table 5 shows the average tSNR scores over all voxels of the tSNR volumes of the original data (i.e., no motion correction applied), and of the retrospectively corrected data through the motion measurements obtained from SLIMM and VVR-LM, respectively, on the patient data set. It can be seen that both the VVR- and SVR-based motion correction methods improved tSNR. It is also evident that, as compared to the VVR-LM method, our SLIMM approach, substantially improved the motion correction performance in terms of tSNR.

In summary, attributed to more accurate motion measurement, our SLIMM approach considerably outperformed the baseline VVR-LM consistently on the real motion data sets in terms of tSNR over the retrospectively corrected data.

### 3.2. Acquisition efficiency and quality

#### 3.2.1. Motion identification

**Fig. 5.**
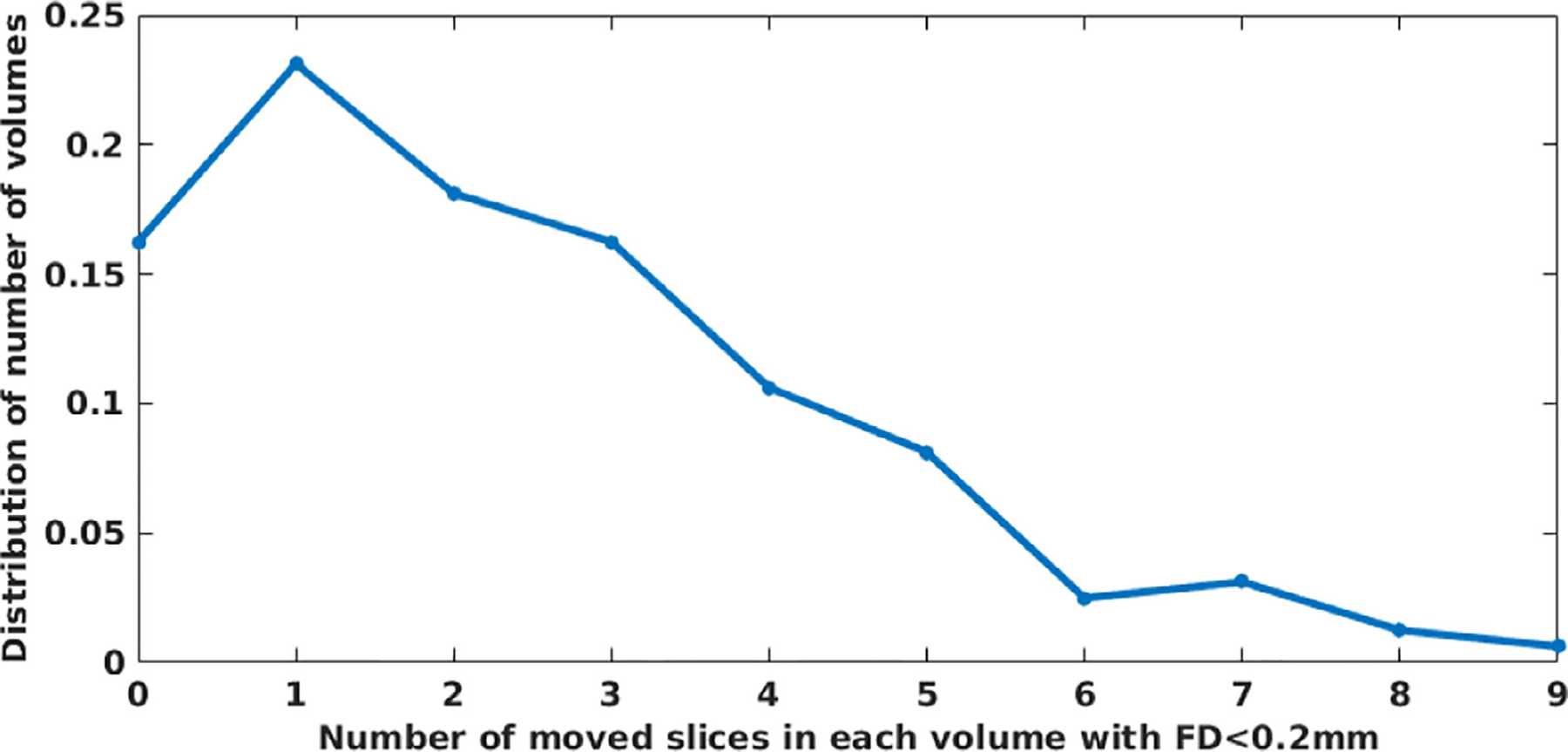
Distribution of the number of volumes over number of moved slices in each volume with FD < 0.2 mm on the EM-Tracking data set. Only 16.5% volumes had all slices displaced less than 0.2 mm, while the rest of the volumes contained at least one slice subject to SD ≥ 0.2 mm. The figure shows that much of the time a volume has an FD < 0.2 mm it still has slices displaced more than 0.2mm.

Fig. 5 shows the distribution of the number of volumes with regard to the number of slices of the volume impacted by motion, considering only those volumes with an FD < 0.2 mm on the EM-Tracking data set. We investigated the SDs measured from over 10,000 slices by using the electromagnetic motion tracking sensor. Only 16.5% volumes had all of its slices exhibit displacement less than 0.2 mm, while the rest volumes contained at least one slice subject to SD ≥ 0.2 mm. This figure shows that even when volumes meet the criterion of FD < 0.2 mm, there are often many slices displaced by over 0.2 mm.

**Fig. 6.**
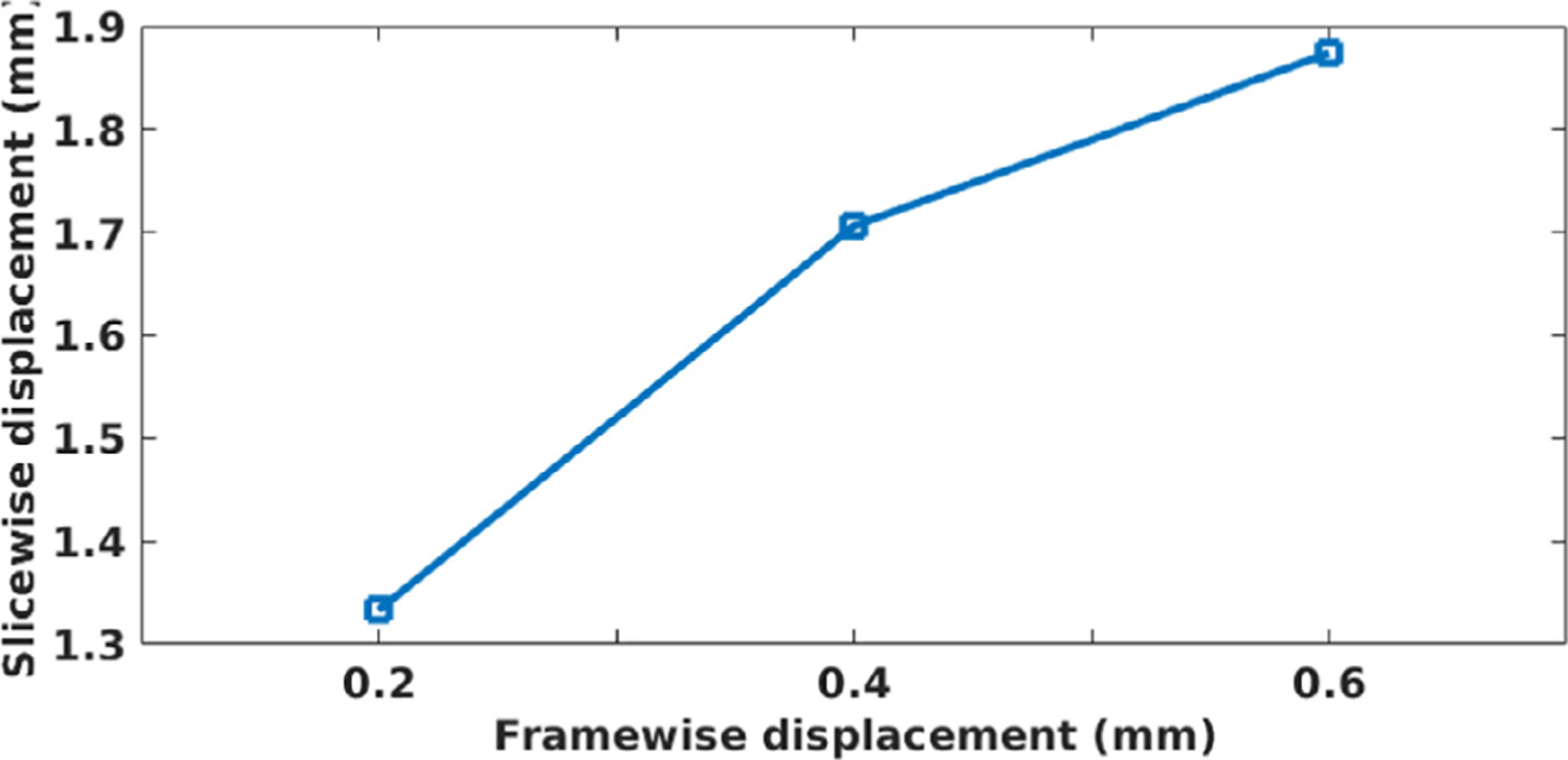
Corresponded threshold values between slice and frame displacements on the EM-Tracking data set. The most widely used threshold on FD is in the range of [0.2,0.6]mm. The corresponding threshold on SD on this data set was between 1.33 mm and 1.87 mm, from high to low. It suggested that VVR-LM was unaware of a moved slice in a volume with FD = 0.2 mm unless this slice displaced at least 1.33 mm.

Fig. 6 shows the corresponded threshold values between SDs and FDs on the EM-Tracking data set. The most widely used threshold on FD ranges from 0.2 mm to 0.6 mm, from high to low. The corresponding threshold on SD on this data set was between 1.33 mm and 1.87 mm, from high to low. This figure shows that VVR-LM was unaware of a moved slice in a volume with an FD = 0.2 mm unless this slice displaced at least 1.33 mm. Considering the widely used FD threshold is between 0.2 and 0.4mm, according to this result, we set the SD threshold to range from one fourth to a half of the slice thickness in all experiments reported in this paper. We were unable to ensure that all slices of a collected volume displaced less than 0.2 mm in VVR-LM with a threshold of 0.2mm on the FD. In contrast, according to our protocol elaborated above, a collected volume by SLIMM guaranteed that all its slices had a motion amount of less than one-fourth of the slice thickness.

#### 3.2.2. Online frame censoring

Table 6 shows the results of experiments with and without motion monitoring on the fMRI sequences of all the data sets that we acquired. Table 7 shows details of the results on the patient data set. Since only 20% of the total number of volumes on each fMRI data sequence were preserved for extending the acquisitions with motion monitoring, there were too many failures in acquiring the desired numbers of motion-free volumes (i.e., over 20% of volumes were excluded from the data collection) when we used the motion threshold of one fourth of the slice thickness for both VVR-LM and SLIMM in this experiment. Therefore, we increased the motion threshold to half of the slice thickness. The lengths of the acquisitions with motion monitoring were shorter than those without motion monitoring. SLIMM took longer acquisitions on average than VVR-LM, since SLIMM performed more accurately in measuring intra-volume motion, and thus successfully identified more volumes containing slices that were subject to motion that VVR-LM overlooked. As a result, SLIMM correctly determined to increase the acquisition lengths. The data quality was improved by excluding all volumes with motion. As shown in Table 6, the tSNR of the motion-corrected data was improved on average by around 88% with SLIMM, and by around 37% with VVR-LM.

#### 3.2.3. Real-time feedback to operator for intervention

**Fig. 7.**
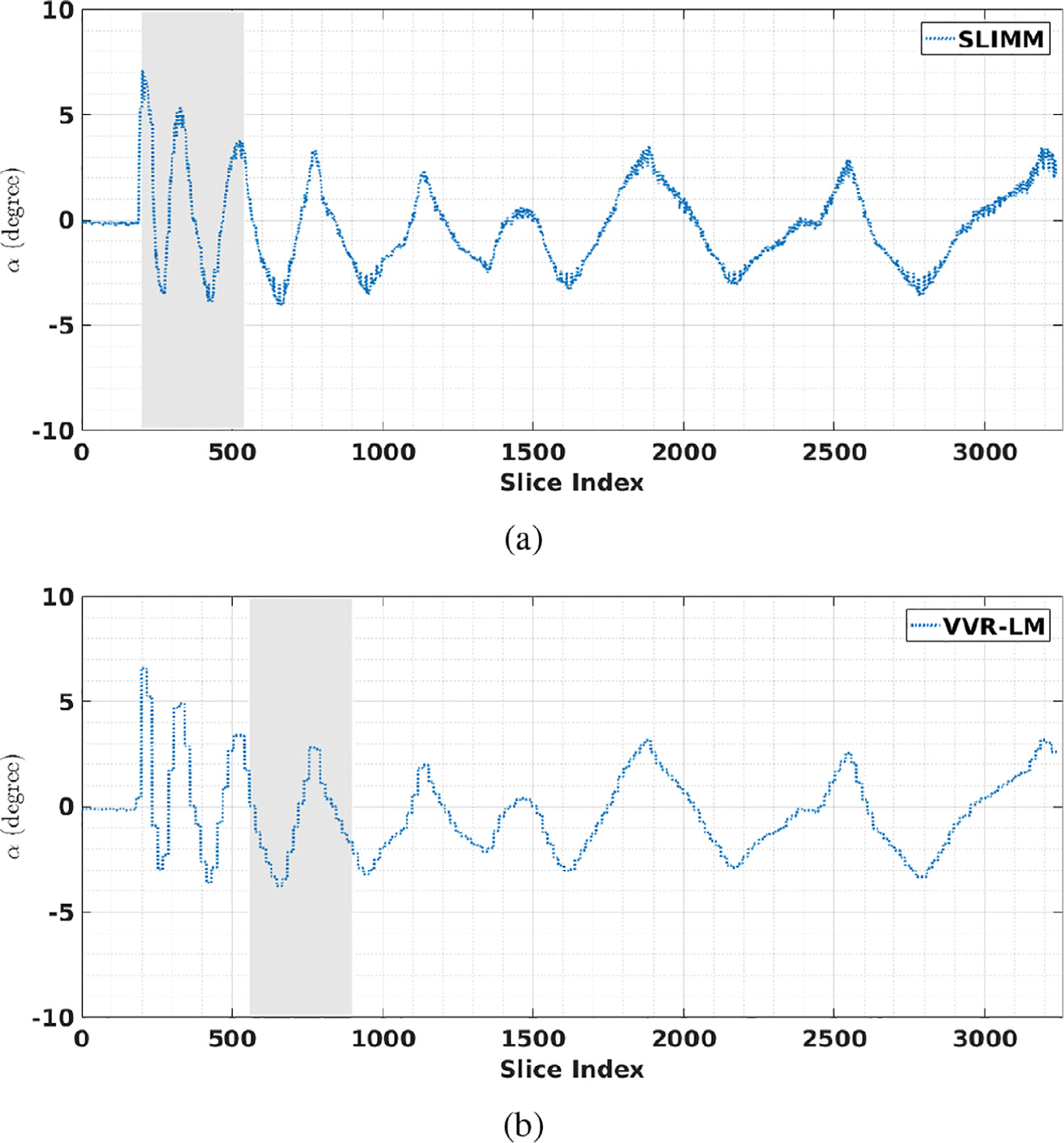
Acquisitions with interventions suggested by the motion monitoring systems of (a) SLIMM and (b) VVR-LM. The gray areas indicate the periods where no motion-free data was collected in the past 30 seconds. The results showed that both motion monitoring systems were able to reduce the scan times by enabling early intervention through their online feedback. SLIMM suggested to intervene in the scan much earlier than VVR-LM, and correspondingly further reduced more scan duration and more associated costs.

Fig. 7 shows the results of the acquisitions with intervention online monitored by SLIMM and VVR-LM, respectively. With a motion threshold of one fourth of the slice thickness, the SLIMM motion monitoring system was aware of the continuous motion when 10 volumes have been acquired, as shown in Fig. 7(a), and suggested operator to intervene in the scan at the 30th volume. In contrast, with the same motion threshold, VVR-LM was unable to trigger the suggestion for intervention. When the threshold was decreased to 0.2 mm, VVR-LM started responding to the continuous motion at the 30th volume, as shown in Fig. 7(b), and suggested intervention when 50 volumes have been acquired. The results showed that both motion monitoring systems were able to reduce the scan times by enabling early intervention through their online feedback. SLIMM suggested intervening in the scan much earlier than VVR-LM, and correspondingly further reduced more scan duration and more associated costs.

### 3.3. Auto-calibration for reference volume

**Fig. 8.**
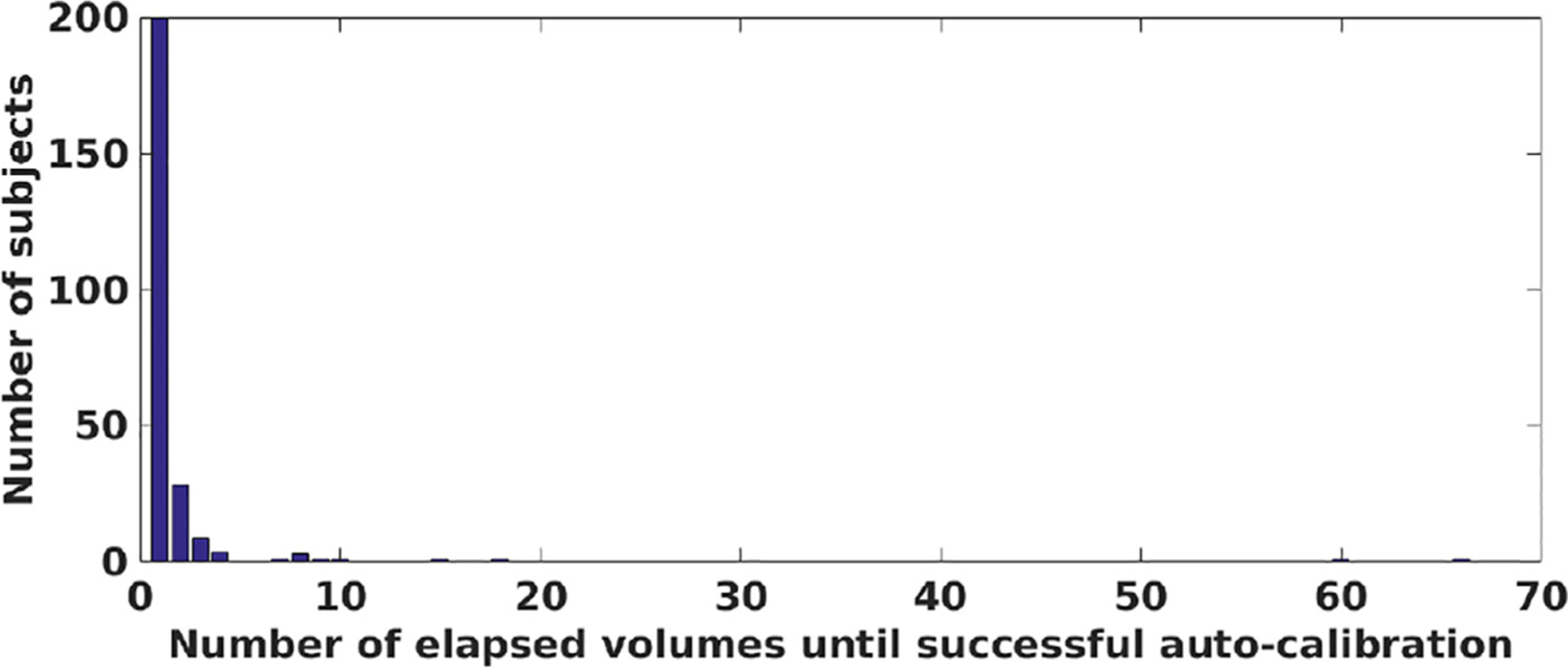
Distribution of the number of subjects over number of elapsed volumes until auto-calibration was successful with a threshold of one-fourth of the slice thickness on the HBN data set. This analysis showed a heavy-tailed distribution with a heavy concentration around 1, which indicated that for the majority of subjects (~ 200 subjects) on the HBN data set, the first volume was successfully chosen as the reference volume; whereas for a few subjects, that apparently moved continuously and significantly, it could take between 15 to 70 volumes before a “motion-free ” reference volume could be obtained. According to our fMRI protocol, for 99.5% of the subjects the auto-calibration time was less than 30 seconds; and only for two cases (among 251 subjects) the auto-calibration took 90–110 s. In addition to showing the efficacy of our auto-calibration method, this analysis is another evidence for the necessity of real-time motion monitoring as it indicates without real-time motion monitoring there is no guarantee that fMRI of sufficient quality is obtained for every subject in a cohort.

As shown in Fig. 8, which shows the distribution of subjects as a function of the number of elapsed volumes until successful auto-calibration. We used a threshold of one fourth of the slice thickness on motion measurements of all slices as an indicator for motion. For about 80% of the subjects on the HBN data set, auto-calibration was completed after the acquisition of the second volume. In these cases the first volume was automatically selected as the reference. The distribution in Fig. 8, with its heavy-tailed shape, also shows that for a few subjects, much more fMRI volumes elapsed until a “motion-free” period was detected to complete the auto-calibration. According to our fMRI protocol a volume can be acquired within 1.5 s with the interleaved and simultaneous multi-slice scheme. With this data, the average time of auto-calibration would be 5.7 s; for 99.5% of the subjects on the HBN data set the auto-calibration time was less than 30 s; and only for two cases (among 251 subjects) the auto-calibration took 90–110 s. In fact, this analysis and the heavy-tailed distribution of the auto-calibration time also provides another evidence for the necessity of real-time motion monitoring to ensure useful fMRI scans are acquired for all subjects within a cohort.

### 3.4. Computational efficiency

We evaluated the SPS of SLIMM on the slices from all the data sets that we acquired. The average SPS was 27.7, i.e., the average time taken in measuring the motion of a slice was ~36 ms. According to our fMRI protocol, it took about 80ms to acquire a simultaneous-2-slice. It thus suggested that SLIMM enabled real-time performance for motion monitoring with our fMRI protocol.

## 4. Discussion

We have developed a real-time SVR algorithm, and applied it to establish a motion monitoring system that we called SLIMM. The interleaved and SMS acquisition schemes have been incorporated in SLIMM. We have conducted extensive experiments to demonstrate the efficacy of SLIMM, and the experimental results have shown that SLIMM led to substantial improvements in the accuracy of motion measurement, and in turn in acquisition efficiency and quality, over the widely-used VVR-based motion monitoring method, resulting in reduced imaging cost and improved data quality.

### 4.1. Motion identification: SVR vs VVR

SVR (VVR) is able to estimate the position and orientation of the subject’s head as the corresponding slice (the volume for VVR) has been acquired, referring to a motion-free volume as the motion reference. Motion can thus be identified from the difference of the motion parameters between the estimate and the reference. In general, SD (FD for VVR) is used to identify motion, which is calculated from the difference of the motion parameters between two consecutively acquired slices (volumes for VVR). Motion happens at the slice level, rather than just at the periods between the acquisitions of consecutive volumes, in the 2D EPI-based fMRI acquisition. By this nature, the VVR-based methods estimate the motion less accurately, and have high temporal delays to be aware of the motion. Since the VVR-based estimate integrates the information from all slices of a volume of interest in the registration, VVR leads to two types of motion identification errors, depending on the time when motion occurs during a single volume acquisition:

- *False positive*. This type of error commonly happens in the case of fast and abrupt motion. As shown in Fig. 3, false positive happened in the acquisition with the VVR-LM motion monitoring system at the period between data points of [200,400] where fast motion occurred. By referring to the optical motion tracking results, VVR-LM had an obvious latency to identify the volumes acquired when fast and abrupt motion happened as motion-corrupted by mistake. Since VVR-LM took all the slices of a real motion-corrupted volume into consideration, it was able to estimate that the volume was corrupted by motion, but the estimated motion amount may be lower than it actually was. This residual led to the false positive error at the next volume, even if the position and orientation of the next volume were accurately estimated.
- *False negative*. This type of error is the major factor to affect the accuracy of VVR, and in particular when frequent motion happened. In the case that the subject moves at the time near the end of a volume acquisition, i.e., only a few slices are corrupted by the motion, the VVR-based monitoring system shows no motion observed, even if the motion amount is high. As shown in Tables 6 and 7, the VVR-LM motion monitoring system yielded slightly higher efficiency in terms of length increment rate than SLIMM, but much lower quality in terms of tSNR. This was caused by the fact that VVR-LM missed to identify the motion and collected by mistake these motion-corrupted volumes.

It is straightforward to perform SVR to estimate the motion in 2D EPI-based fMRI acquisitions. Our protocol for collecting data is that, a volume would be excluded, if any of its slices are motion-corrupted. As a result, SLIMM leads to the above errors at a very low probability, i.e., only when intra-slice motion happens. Considering the EPI slices are acquired very fast (60–80ms), the above errors can be negligible in SLIMM. This is the major reason that SLIMM substantially improved the monitoring efficiency and data quality, and considerably decreased the temporal monitoring delay, as compared to VVR-LM.

### 4.2. Displacement: frame vs slice

The frame displacement (FD) (Power et al., 2012) is computed by the sum of absolute head movement in all six rigid body directions from two consecutively acquired volumes. Similar definitions are possible, but the precise nature of the measure is not critical to the overall purpose of characterizing motion. For example, a Euclidean distance could be used in place of the Manhattan distance described above, and a chord length could be used instead of an arc length. Let **T**_*i*−1_ and **T**_*i*_ be consecutive rigid body transforms aligning each of the consecutive volumes number (*i* − 1) and *i* to a reference volume. The transform that describes the change in position and orientation between these two volumes is then **T** = **T**_*i*−1_ (**T**)^−1^, the composition of the transform aligning the volume at the prior position to the reference volume, with the transform from the reference volume to the current position. Other measures of displacement using this composed transformation are possible.

We have generalized the FD to slices, as denoted by the slice displacement (SD), by considering the possibility that each slice, not just each volume, may undergo an independent rigid body transformation. We have used the SD measure for monitoring motion occurring at the slice level. When imaging subjects, the head may undergo rigid body motion at any time during the slice readout, not only at the times that are instants between different volumes. Consequently, although rigid body motion is assumed for the volumes in order to calculate the FD, the motion of the collection of slices in the volume cannot always be described as a single rigid body motion. Instead, the possibility of rigid body motion at each slice must be considered. The observed motion of the slices *j* ∈ {1, … *, n*} of the volume *i* is then *SD*_*ji*_ with *n* being the number of slices per volume. The sum of the displacements of each slice of a volume is then a measure of the total displacement during the volume acquisition, but this is not equal, in general, to the FD which assumes one rigid body transform describes the motion of all of the slices: $\sum_{j}^{n}SD_{ji}\neq FD_{i}$.

Consequently, the FD measure represents the displacement of an overall rigid body transformation that is estimated by the rigid registration when one or more of the slices may be displaced by different rigid body transformations. In contrast, our SD measure reflects the change in position of the slice that undergoes rigid body transformation, which is easy to interpret.

Note also that if the slice is not encoded correctly, or there is motion during the acquisition of calibration lines used to compute the GRAPPA kernel, or coil sensitivity profiles for SENSE, severe signal intensity artifacts may be present, and such slices or volumes should be dropped from consideration rather than used in the computation of an alignment.

### 4.3. Motion threshold on slice displacement

The most widely used protocol in motion monitoring is to impose a motion threshold on FD (Power et al., 2012). If a volume has an FD less than the threshold, it is considered as motion-free and accepted to be collected; otherwise, the volume is regarded as motion-corrupted and excluded from the data collection (frame censoring) (Ciric et al., 2017). In general, the threshold is set between 0.2 and 0.4mm from high to low. Using a higher threshold is more sensitive to small motion, and excludes more volumes during the monitored acquisition, leading to longer scans but higher data quality. Using a lower threshold tolerates more significant motion, and thus collects more volumes with low movements, resulting in shorter scans but lower data quality. As a result, it is a trade-off to set a motion threshold for the monitored acquisition between motion sensitivity and motion tolerance.

In motion monitoring system at the slice level, subject motion is measured slice by slice. Therefore, SD is leveraged, instead of FD, to identify if a slice displaces. Our protocol used in SLIMM was to exclude a volume if this volume contained any motion-corrupted slices; and otherwise, to collect it.

As addressed above, while it has been determined empirically that an FD of between 0.2 mm and 0.4 mm indicates the amount of motion that can be tolerated during an fMRI experiment, this numerical value cannot be used for SD, because the FD represents the displacement of an overall rigid body transformation that is estimated by the rigid registration when one or more of the slices may be displaced by different rigid body transformations. We set the threshold on SD at one fourth and half of the slice thickness, from high to low. This setting was much lower than that used for FD thresholding in the numerical value. However, it did not indicate that we tolerated significant motion much more in the experiments. We have demonstrated that one fourth of the slice thickness is high enough to the SD thresholding, since a volume was examined slice by slice under this criterion. In fact, it was unable to ensure that all slices of a collected volume displaced less than 0.2 mm in VVR-LM with a high threshold of 0.2mm on the FD. A slice may displaced up to 1.33 mm within a volume of FD less than 0.2 mm according to our results that have shown in Fig. 6. In contrast, a collected volume by SLIMM was guaranteed that all its slices had a motion amount of less than one fourth of the slice thickness. This was also the reason that SLIMM performed with more accurate motion measurement than VVR-LM. For consistency, we used the same motion threshold of one fourth of the slice thickness in the experiments for both SLIMM and VVR-LM. Such a setting was preferred by VVR-LM for low acquisition duration in the experiments, such as those reported in Section 3.2. Although it was unfair to SLIMM with this threshold setting, the experimental results have still shown that SLIMM considerably outperformed VVR-LM consistently on various data sets.

### 4.4. Motion monitoring: SLIMM vs VVR-LM

As shown in the experiments reported in Section 3.2, SLIMM acquired more volumes than VVR-LM, while achieved much higher improvement in data quality in terms of tSNR. During the acquisitions, SLIMM correctly identified that there were slices that were subject to motion that VVR-LM overlooked. Consequently, SLIMM was able to correctly recommend increasing the scan duration to account for the motion-corrupted data that was incorrectly missed by VVR-LM, since VVR-LM examined only volumes. As a result, motion monitoring (with either SLIMM or VVR-LM) led to fewer actually acquired volumes and thus reduced the scan duration compared to acquisitions with no motion monitoring, and SLIMM led to much higher data quality (according to tSNR) than VVR-LM.

If the subject does not move at all, and the VVR-LM user knows ahead of time that the subject will not move, then head motion is not a concern, and both SLIMM and VVR-LM may lead to the same quality of data and the same minimum scan time. In practical fMRI acquisitions, however, head motion is the single most important confounder in fMRI studies, and is common and widespread. Consequently, it is desirable to mitigate against the possibility of motion.

If there is intra-volume slice motion, then SLIMM will indicate the need to acquire additional data, and VVR-LM may fail to detect the motion, and falsely indicate there is no problem, when there is a problem. This leads to worse tSNR with VVR-LM. If there is intra-volume slice motion, and detectable volume-to-volume motion, then again SLIMM will indicate the need to acquire additional data, and VVR-LM will indicate the need to acquire some additional data. Since VVR-LM is insensitive to some motion, it may fail to fully signal the true extent of motion, leading to a worse tSNR with VVR-LM in addition to extended scan time.

When the designer of an fMRI experiment considers monitoring motion with VVR-LM and acknowledges that 1) motion is common and 2) VVR-LM does not detect all the motion, and 3) reduced tSNR is bad for data analysis, then they may prefer to mitigate against the possibility of motion, and consequent data quality loss, by acquiring additional data. Extending the scan time may lead to the capture of sufficient data to compensate for the tSNR loss that occurs with VVR-LM motion monitoring. Thus, the VVR-LM user chooses to extend their scan time beyond the minimum, in order to mitigate against the possibility of unrecognized head motion, and to restore some lost tSNR (without knowing how successful they will be). In comparison, the SLIMM user who does not know if their subject will move or will not move, does not need to extend the scan time in case there is unmonitored motion because all the motion is monitored, and can use a shorter scan time to achieve the desired tSNR. The VVR-LM users who is certain that their subjects will either not move at all, or will not exhibit unmonitored motion, are able to achieve the same short scan time and sufficient tSNR as SLIMM. However, in designing practical fMRI acquisitions, it is usually not possible to know before imaging that subjects will not move or will not exhibit motion that VVR-LM is insensitive to. As a result, a designer planning to use VVR-LM for motion monitoring instead of SLIMM should choose to run for a longer scan time in order to expect to achieve the same desired level of tSNR.

In addition, both the motion monitoring systems were able to provide real-time feedback to the operator to enable immediate intervention for the scans. Therefore, the scan times and the associated costs were substantially reduced. Moreover, SLIMM suggested intervening in the scan much earlier than VVR-LM for the acquisitions with continuous subject motion, since SLIMM was able to correctly identify the motion-corrupted slices that VVR-LM overlooked during the monitoring. It indicated that SLIMM further saved more scan time and more associated costs than VVR-LM.

### 4.5. SVR: single-slice vs SMS

Our SLIMM algorithm can be used with sequences where one single slice is acquired at a time, instead of using SMS. The potential advantage of SMS is that two or more slices maximally separated across a volume of n slices have a more accurate and stable registration optimum than one slice alone. One slice alone may even be outside of the brain, or contain a very small amount of brain tissue at the edge of the brain, which may lead some slices to have poor alignment. This type of error can be tolerated when doing motion monitoring, but it can be mitigated by using more than one slice for the motion monitoring even when a single slice at a time is acquired. This leads to a slower update rate for the motion monitoring, but remains much faster than volume to volume motion monitoring.

### 4.6. Insights of auto-calibration for reference volume

In the demonstration of our auto-calibration method for the reference volume, experimental results in a large cohort of pediatric subjects in Fig. 8 showed that our auto-calibration method quickly found a reference volume for the majority of subjects. In a few cases with significant motion, it took a relatively long time. This analysis showed the efficacy of our auto-calibration method, and also showed the importance of motion monitoring during fMRI acquisitions. It indicated that we cannot just rely on a long acquisition protocol and expect to get sufficient data for all cases in a cohort. Without real-time motion monitoring, we may end up scanning a large number of pediatric subjects for unnecessary long scan time, and yet not be successful in acquiring useful data for some subjects. These issues with subject motion become more prominent when studying and scanning non-cooperative patient populations such as infants, toddlers, and young children. With real-time motion monitoring and an adaptive strategy for extending the length of fMRI scans, until a desirable number of “motion-free ” periods are collected, we can ensure that an fMRI protocol runs efficiently for a cohort of patients and subjects as well as for an individual in critical condition scanned in a clinical setting, e.g., for presurgical planning. Therefore, real-time motion monitoring results in a substantial reduction in average scan times, reduces the burden of long acquisitions on patients, and reduces the costs and delays associated with repeated acquisitions. Our approach (SLIMM) is a cost-efficient and safe, self-navigated, fast, slice-level motion monitoring system, that does not require any external hardware attachment or pulse sequence modification, therefore it can be safely and easily used with different fMRI paradigms for pediatric and non-cooperative patients.

### 4.7. Conclusion

Two major challenges for constructing a real-time slice-by-slice motion monitoring system are 1) to ensure high accuracy of motion measurement, and 2) to perform the monitoring in real-time with the ultrafast 2D EPI fMRI acquisition. In this work, we developed and presented a real-time slice-to-volume image registration algorithm with a parallelized patch-based approach. Based on this registration algorithm, a motion monitoring system, named SLIMM, was established for fMRI. Extensive experiments on real fMRI data sets with real motion demonstrated that SLIMM performed accurately, robustly, and in real-time for slice-by-slice fMRI motion monitoring. Extensive experimental results reported here have shown that 1) motion monitoring is critical for efficient and high quality fMRI acquisition; 2) SLIMM outperformed VVR-LM (a VVR-based approach which is currently the most widely used method in fMRI motion monitoring) in terms of both accuracy of motion measurement and data acquisition efficiency; 3) using both interleaved and simultaneous multi-slice acquisition schemes improved robustness of the SVR; 4) our patch-based matching criterion improved both the accuracy and running speed of the SVR; 5) SLIMM ensured that the amount of acquired fMRI data was sufficient for the desired analysis, while not spending time on acquiring data that was in excess of what was required, i.e., scanning-to-criterion, leading to considerable improvement in motion measurement accuracy and substantial reduction in scan times and the associated costs; and 6) SLIMM provided real-time feedback to the scanner operator and the subject to enable further motion reduction, and correspondingly further reduced the scan duration.

## Figures and Tables

**Table 1:** Description of the fMRI time series obtained using 2D gradient-echo EPI sequences on different data sets.

Data Set	#Measurements	Interleave Factor	SMS Factor	TR/TE (ms)	Slice Thickness (mm)	#Slices	Matrix Size
*EM-Tracking*	96	2	2	1500/30	3	36	64 × 64
*Optical Motion Tracking*	180	2	2	1500/30	3	36	64 × 64
*Patient*	160 / 96	2	2	1500/30	3	36	72 × 72

**Table 2:** Mean and standard deviation of motion measurement errors in terms of motion transformation parameters and SDs obtained from SLIMM and VVR-LM methods on the EM-Tracking data set. Our method, SLIMM, offered sub-voxel slice-level accuracy in this difficult task.

	Translation (mm)	Rotation (degree)	Displacement (mm)
VVR-LM	1.17 ± 1.20	1.64 ± 3.00	3.14 ± 4.36
SLIMM	0.71 ± 0.64	0.77 ± 1.03	1.37 ± 1.81

**Table 3:** Mean and standard deviation of the motion measurement errors in SD obtained from SLIMM and VVR-LM methods on the optical motion tracking data set. Our methods, SLIMM, offered sub-voxel slice-level accuracy in this difficult task. The difference in performance which showed the advantage of SLIMM was very large for fast motion.

	Overall (mm)	Fast Motion Period (mm)
VR-LM	0.77 ± 0.94	3.12 ± 2.24
SLIMM	0.32 ± 0.22	0.44 ± 0.37

**Table 4:** Average tSNR scores on all voxels of the tSNR volumes on the EM-Tracking data set with the frame censoring on (shown without parentheses) and off (shown in parentheses). The best results are highlighted by the bold-face font.

	Reference scans		Evaluation results	
	Raw-No-Motion	Raw-Motion	VVR-LM	SLIMM
Subject 1	61.56 (63.32)	8.16 (6.93)	18.77 (11.83)	**27.49** (17.63)
Subject 2	62.63 (62.67)	17.59 (16.12)	32.47 (24.69)	**46.11** (36.52)
Subject 3	65.94 (65.50)	14.55 (14.43)	14.80 (14.45)	**51.26** (36.10)
Subject 4	44.18 (42.56)	10.88 (10.13)	27.85 (20.22)	**37.86** (28.13)
Subject 5	81.42 (76.53)	11.86 (10.82)	15.08 (13.29)	**30.10** (23.51)
Subject 6	70.97 (69.70)	10.80 (10.14)	10.80(10.14)	**27.44** (21.62)
*mean*	64.76 (63.72)	12.33 (11.45)	19.87 (15.71)	**36.83** (27.28)

**Table 5:** Average tSNR on all voxels of tSNR volumes on the patient data set. The best results are highlighted by the bold-face font. Compared to VVR-LM, our method, SLIMM, substantially improved the tSNR on all 3 patient data sequences.

	Original data	VVR-LM	SLIMM
Patient #1 (resting state)	35.89	40.72	**47.29**
Patient #2 (resting state)	52.23	55.48	**62.81**
Patient #3 (finger tapping)	28.21	30.17	**35.45**

**Table 6:** Analysis results of the two motion monitoring methods on all data sets that we acquired with the motion threshold of half of the slice thickness on SD (for SLIMM) and FD (for VVR-LM). Both methods reduced the scanning duration; but SLIMM led to much higher data quality (according to tSNR) compared to VVR-LM at the cost of only marginally longer scans (measured by the length increment rate).

Length increment rate			tSNR gain		
No Monitoring	VVR-LM	SLIMM	No Monitoring	VVR-LM	SLIMM
25%	16.67%	17.86%	-	37.15%	88.04%

**Table 7:** Analysis results of the two motion monitoring methods on the patient data set with the motion threshold of half of the slice thickness on SD (for SLIMM) and FD (for VVR-LM). Compared to no motion monitoring and VVR-LM, SLIMM collected the minimum necessary amount of data (based on the predefined criteria), leading to improved data quality (in terms of tSNR) and reduced scanning time (in terms of number of actually acquired volumes).

	# of volumes actually acquired				tSNR score		
	Expectec	No Monitoring	VVR-LM	SLIMM	No Monitoring	VVR-LM	SLIMM
Patient #1 (resting state)	128	160	133	135	35.89	43.96	47.11
Patient #2 (resting state)	128	160	128	128	52.23	61.09	70.01
Patient #3 (finger tapping)	76	96	79	80	28.21	31.65	35.67
